# Engineering the Electrical and Optical Properties of WS_2_ Monolayers via Defect Control

**DOI:** 10.1002/advs.202305162

**Published:** 2023-11-27

**Authors:** Michele Giovanni Bianchi, Francesca Risplendi, Michele Re Fiorentin, Giancarlo Cicero

**Affiliations:** ^1^ Department of Applied Science and Technology Politecnico di Torino corso Duca degli Abruzzi 24 Torino 10129 Italy

**Keywords:** defect engineering, intrinsic defects, opto‐electronic properties, substitutional defects, surface charge transfer doping

## Abstract

Two‐dimensional (2D) materials as tungsten disulphide (WS_2_) are rising as the ideal platform for the next generation of nanoscale devices due to the excellent electric‐transport and optical properties. However, the presence of defects in the as grown samples represents one of the main limiting factors for commercial applications. At the same time, WS_2_ properties are frequently tailored by introducing impurities at specific sites. Aim of this review paper is to present a complete description and discussion of the effects of both intentional and unintentional defects in WS_2_, by an in depth analysis of the recent experimental and theoretical investigations reported in the literature. First, the most frequent intrinsic defects in WS_2_ are presented and their effects in the readily synthetized material are discussed. Possible solutions to remove and heal unintentional defects are also analyzed. Following, different doping schemes are reported, including the traditional substitution approach and innovative techniques based on the surface charge transfer with adsorbed atoms or molecules. The plethora of WS_2_ monolayer modifications presented in this review and the systematic analysis of the corresponding optical and electronic properties, represent strategic degrees of freedom the researchers may exploit to tailor WS_2_ optical and electronic properties for specific device applications.

## Graphical abstract

Controlling the electric and optical properties of WS_2_ monolayers via intentional defect inclusion is an effective way to mitigate the intrinsic defect issue and tailor the material properties. This work provides a systematic analysis of defects in WS_2_, considering both intrinsic defects and different, innovative doping schemes, reporting a combination of theoretical and experimental evidence.

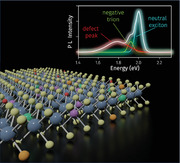

## 1 Introduction

2D materials such as transition metal dichalcogenide monolayers (TMD MLs) emerged as ideal candidates for innovative electronic and optoelectronic devices, since they intrinsically push the scaling process down to the atomic level, overcoming common technological limits.^[^ ^1^, ^2^, ^3^, ^4^ ^]^ Among the plethora of TMDs, direct‐bandgap semiconductors such as WS_2_ MLs in the 1H phase, have been studied due to their unique combination of atomic‐scale thickness, optoelectronic, and mechanical properties, that make them suitable for a wide range of applications.^[^ ^5^ ^]^ Recently, significant efforts have been dedicated to increasing the size of the MLs from tens of μm up to the wafer scale^[^ ^6^, ^7^ ^]^ and improving the compatibility of the ML synthesis with the standard semiconductor technology processes.^[^ ^8^, ^9^ ^]^ In this regard, chemical vapor deposition (CVD) has emerged as the only promising synthesis route for large‐scale production, since other approaches such as exfoliation or epitaxial growth are not scalable or result in small and irregular samples.^[^ ^10^ ^]^ At the same time, the main limit of CVD is the poor crystalline quality of MLs due to the abundance of defects introduced during the synthesis that can significantly worsen the material properties.^[^ ^11^ ^]^ Acknowledging the inevitability of defects, current technological advancements strive to achieve comprehensive and intentional control over them. Rather than regarding defects as drawbacks, they can be harnessed as valuable resources to manipulate, enhance, and introduce novel properties to MLs. Defect engineering proves to be remarkably effective in 2D materials due to their heightened susceptibility to structural defects compared to their bulk counterparts. This susceptibility arises from the reduced electrostatic screening in monolayer systems,^[^ ^12^ ^]^ making defect engineering an indispensable tool for tailoring the properties of WS_2_ ML.

A wide range of worthy reviews about defects in TMDs are already available, even though they mainly focus on 1H‐MoS_2_, while only reporting sparse examples about other materials.^[^ ^13^, ^14^ ^]^ Hence, this review is totally devoted to the analysis of defects in 1H‐WS_2_, a TMD with promising superior properties with respect to the extensively studied MoS_2_. The strength of WS_2_ resides in its applicability in optoelectronic devices able to emit in the visible range due to the larger energy gap of WS_2_ (*E*_g_ = 2.05 eV^[^ ^15^ ^]^) with respect to MoS_2_ (*E*_g_ = 1.8 eV^[^ ^16^ ^]^). Moreover, the photo‐luminescence (PL) quantum yield in WS_2_ MLs is about 20 times larger than in MoS_2_ ones,^[^ ^17^ ^]^ making WS_2_ a superior material for emitting devices. In addition, WS_2_ MLs are characterized by higher mobilities with respect to other TMD MLs, resulting in superior transistor performances.^[^ ^18^, ^19^ ^]^

Differently from other reviews that focus only on a specific defect engineering approach (e.g., substituent defects,^[^ ^20^ ^]^ defects at the chalcogen site,^[^ ^21^ ^]^ vacancies^[^ ^22^ ^]^), here we systematically report examples for all the common defects discussed in the literature for WS_2_.

The structure of this review is organized as follows: Section 2 provides a brief discussion on the PL spectra of WS_2_ ML. This section aims to help non‐expert readers gain a better understanding of the modification of the PL due to defects reported in the next sections. Section 3 briefly resumes the common as well as the most advanced characterization techniques employed in the study of defects in 2D materials. Then, intrinsic defects are analyzed since, even at low concentrations, they modify the WS_2_ properties due to the enhanced influence of defects in 2D materials (Section 4). Following, different doping techniques, such as substituents at the tungsten (Section 5.1) and at the sulfur (Section 5.2) sites, as well as surface charge transfer doping (SCTD) by atom (Section 5.3) and molecule (Section 5.4) adsorption, are analyzed in view of the control of WS_2_ electronic and optical properties. For each case, starting from ab initio simulations, the variation of the band diagram due to the defects is discussed and correlated to the electric‐transport measurements in field‐effect transistors (FETs). Then, the modulations of the electronic properties by defects are associated to the optical properties of the MLs, mainly analyzing the PL spectra. The goal is to provide a complete view of each analyzed defect. Finally, possible future research trends in defect engineering in WS_2_ are proposed (Section 6). Globally, we depict a large overview of the vast array of possibilities for tuning the WS_2_ ML properties by defect engineering, assisting future studies in selecting the most appropriate approaches for tailoring WS_2_ in various scenarios.

## 2 Analysis of the Photo‐Luminescence Spectrum of WS_2_ Monolayer

**Figure 1:**
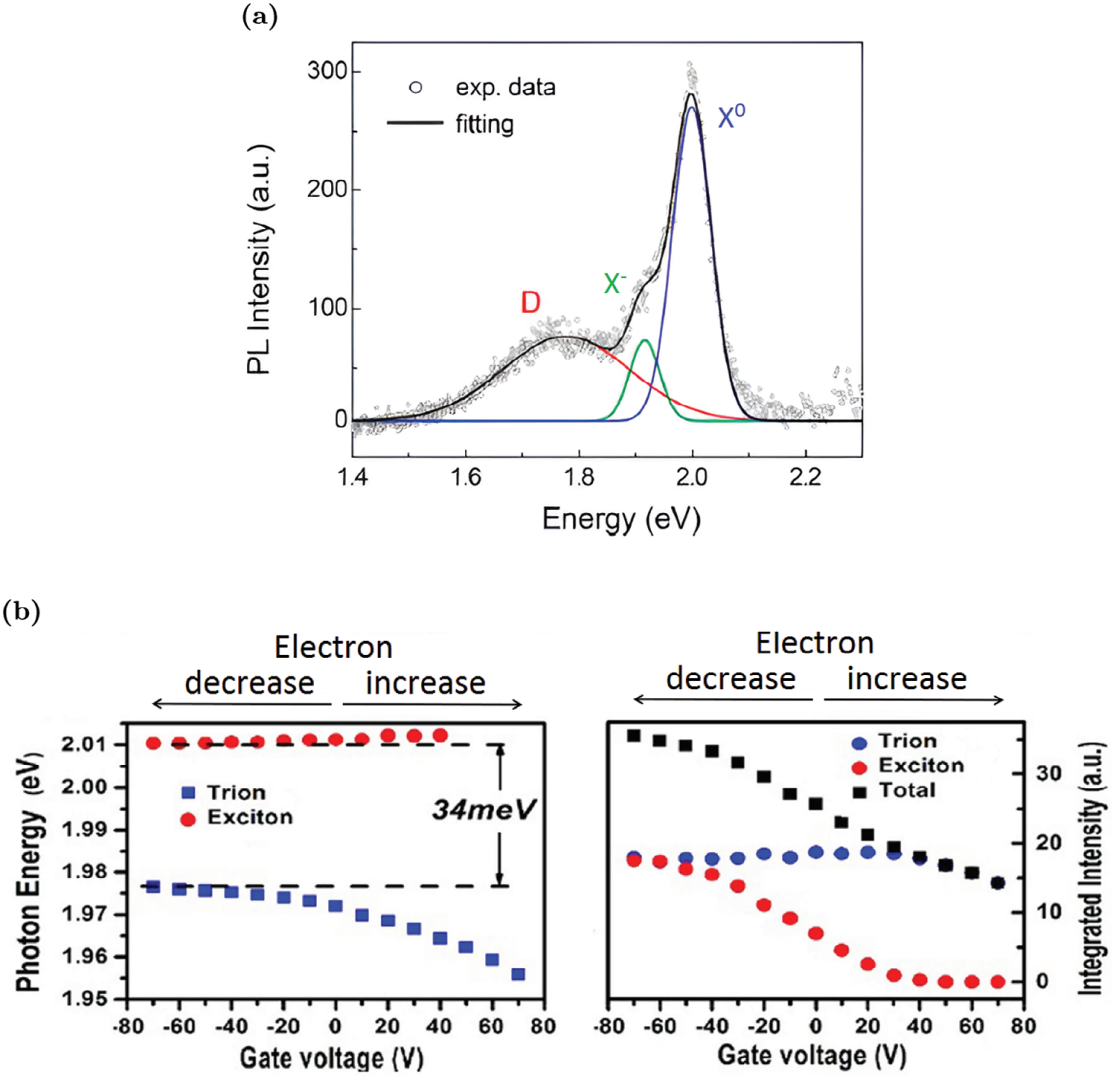
Overview of PL properties of WS_2_ MLs. a) PL spectrum at 77 K. Adapted with permission.^[^ ^25^ ^]^ Copyright 2019, American Chemical Society. b) Room temperature PL peak position and intensity dependence on electron concentration. Electron modulation is achieved by electro‐doping in FET‐WS_2_ ML: Positive (negative) voltages are responsible for an increase (decrease) of the electron concentration. Adapted under the terms of the CC‐BY 4.0 license.^[^ ^26^ ^]^ Copyright 2015, The Authors, Published by Springer Nature.

This section provides a brief summary of the interpretation of the PL spectra of WS_2_ MLs and serves as a starting point for the subsequent discussions on the modifications of WS_2_ optical properties in different contexts. For a more complete discussion about PL spectra, interested readers can refer to specific works.^[^ ^23^, ^24^ ^]^ An example of a typical PL spectrum of WS_2_ ML at low temperatures is reported in **Figure** **1**a. The interpretation of the PL spectra in WS_2_ MLs is a challenging task due to the presence of various neutral and charged exciton states. The weak dielectric screening in ML enhances the Coulomb interaction, making different exciton species stable even at room temperature.^[^ ^23^ ^]^ The most significant peak in the spectrum in Figure 1a, X^0^, is associated with the neutral exciton, a bound state of an electron and a hole, interacting via electrostatic Coulomb force. Since electrons in this state are interacting with holes, their ground state energy is slightly smaller than the “quasi‐free” electron energy in the conduction band. For that reason, the optical transition associated with an exciton recombination occurs at energies smaller than the nominal electronic gap. The precise energy position of the PL peak associated to the neutral exciton recombination depends on different factors such as crystal quality, carrier concentrations, temperature, and laser excitation power. However, it is about 2 eV at room temperature.^[^ ^23^, ^24^ ^]^ In addition to the neutral exciton, charged excitons (i.e., bound states composed of three interacting charge carriers), also called trions, are commonly observed in WS_2_ MLs. Due to the n‐type nature of WS_2_ MLs, negative trions composed of two electrons and a hole are more likely.^[^ ^23^ ^]^ The PL peak associated to the optical recombination of negative trions (X^−^ in Figure 1a) falls at smaller energies than the neutral exciton one. Its position can vary from about 1.92 to 1.98 eV.^[^ ^25^, ^26^ ^]^ Indeed, the positions and intensities of both the X^0^ and the X^−^ peaks markedly depend on the electron concentration.^[^ ^24^ ^]^ The increase in the electron concentration causes an increase in the electrostatic screening between free electrons and holes, reducing the formation probability of the exciton species. However, at the same time, a higher electron concentration favors the negative trion formation, providing the second electron for the trion.^[^ ^27^ ^]^ For that reason, when the electron concentration rises, it leads to a reduction in the overall PL intensity. However, there is a relative enhancement of the negative trion peak compared to the neutral one. Globally, there is a red‐shift of the PL emission due to the exciton‐to‐trion conversion.^[^ ^24^ ^]^ On the contrary, the decrease in the free electron concentration is responsible for a PL enhancement together with a blue‐shift due to the trion‐to‐exciton conversion. In addition to the variations in peak intensity, the position of the individual peaks also depends on the carrier concentration. Notably, the negative trion peak shows a red‐shift as the electron concentration rises.^[^ ^24^ ^]^ These modifications of the PL peak intensities and positions due to variations in carrier concentration are summarized in Figure 1b. Understanding these trends is crucial, as they serve as key points for explaining the PL variations induced by defects in WS_2_ MLs.

In addition to the neutral and negative exciton peaks, there are other PL peaks that can be identified at low energies, such as defect‐bound excitons and biexcitons.^[^ ^23^ ^]^ For the purpose of this review, we will focus specifically on the defect‐bound exciton peak (D in Figure 1a). A defect‐bound exciton is an exciton localized close to a defect site. The presence of defect‐bound excitons is associated with defects with peculiar deep in‐gap states capable of trapping charge carriers. For that reason, the defect‐bound exciton peak appears at lower energy compared to the peaks corresponding to the neutral or charged excitons. However, the defect‐bound exciton peak is visible mainly at cryogenic temperatures since the thermal excitation is sufficient to release the defect‐bound exciton and convert it into a free exciton. Hence, the analysis of defect‐bound exciton peaks in low‐temperature PL measurements serves as an approach to assess the crystalline quality of MLs.^[^ ^28^ ^]^

Another interesting optical feature of WS_2_ MLs is the evidence of nonlinear optical (NLO) effects, that is, phenomena in which the induced polarization due to light‐matter interaction nonlinearly depends on the external electrical field.^[^ ^29^ ^]^ Different NLO effects are observed in TMD MLs, however the most commonly reported phenomenon is second harmonic generation (SHG), which can exist in MLs due to the breaking of the inversion symmetry with respect to the bulk systems.^[^ ^29^ ^]^ Interested readers can refer to specific reviews for a more complete description of all NLO effects in 2D materials.^[^ ^29^, ^30^, ^31^ ^]^ In SHG, two photons with the same frequency interact with the nonlinear material and are converted to a photon with doubled frequency. The absorption of photons with energy smaller than the energy gap is mediated by virtual states.^[^ ^31^ ^]^ However, the presence of additional states in the gap due to defects can alter this absorption process.^[^ ^32^ ^]^ The robustness of SHG in WS_2_ ML was tested considering different types of defects, including intrinsic defects, intentionally created damages, and adsorbed molecules. It results that SHG emission tends to be more resilient in the presence of defects compared to PL. SHG is suppressed only in case of large and intentionally created defects.^[^ ^33^ ^]^ In some cases, the presence of defects is even able to enhance SHG, while the PL is markedly suppressed.^[^ ^32^ ^]^ The effects of defects on the SHG are discussed in more detail for specific cases in the following sections.

## 3 Characterization Techniques for Defects

This section provides some basic information about the characterization techniques employed to analyze defects. Our description is not intended to be exhaustive, but it aims at revisiting the main features of the methods used to investigate the electrical and optical properties of defective WS_2_ MLs. For a more complete description of the characterization techniques in 2D materials, the interested readers can refer to specific works.^[^ ^34^, ^35^, ^36^, ^37^ ^]^ The characterization techniques discussed in this section are outlined in **Table** **1**, which highlights the key information they offer. Some of these approaches are commonly applied in different fields and are not extensively discussed; instead, reference examples in Table 1 illustrate their practical use for studying defects in WS_2_ MLs. On the contrary, more detail is provided for less conventional techniques that offer valuable insights into understanding defects in WS_2_. Among the most versatile techniques, the category of scanning probe microscopy (SPM) techniques stand out for their ability to locally excite defect sites and investigate their properties with nanometer‐scale spatial resolution. Scanning tunneling microscopy (STM) is the typical example of an SPM approach that allows to map the WS_2_ ML surface with atomic resolution.^[^ ^38^, ^39^ ^]^ By applying a constant voltage and measuring the tunneling current between the WS_2_ and a conductive tip (fraction of nanometres far from the sample), it is possible to reconstruct the surface morphology up to the atomic roughness. Hence, defects can be easily visualized and different defects can be distinguished due to their own electronic fingerprint visible in the STM maps.^[^ ^38^ ^]^ Using the same STM setup, but applying a variable voltage between the sample and the tip, it is possible to measure the current–voltage characteristic of the tip‐sample system (scanning tunneling spectroscopy [STS] mode). The associated differential conductance directly gives access to the evaluation of the local density of states LDOS (i.e., the number of electronic states at a specific energy per unit of volume of the sample in proximity of the tip).^[^ ^40^ ^]^ Hence, an STS measurement on a defect site provides a direct picture of the electronic properties and uniquely defines the defect type. For that reason, STS spectra, compared to ab initio simulated LDOS, are a powerful tool to identify the nature of unknown defects in WS_2_, as shown in Section 4.^[^ ^41^ ^]^ Atomic force microscopy (AFM) is another technique adopted to study the surface morphology of WS_2_ ML.^[^ ^42^ ^]^ However, a standard AFM setup has insufficient spatial resolution to visualize single defects. Only AFM in non‐contact mode, and using a tip functionalized with a CO molecule on the apex, is able to reach true atomic resolution.^[^ ^43^, ^44^ ^]^ This technique allows to clearly identify the defect site without electronic artifacts often present in STM maps and it is also able to distinguish between defects in the top and bottom sulfur plane of the WS_2_ ML.^[^ ^38^ ^]^ Another relevant technique for the study of defects in WS_2_ ML is tip‐enhanced Raman spectroscopy (TERS).^[^ ^45^, ^46^ ^]^ Standard Raman spectroscopy offers valuable insights into defects, but its spatial resolution is constrained to the range of hundreds of nanometers due to optical diffraction limitations.^[^ ^45^ ^]^ In TERS setups, the use of a metallic SPM tip, in proximity of the probing site, allows to enhance the Raman signal due to plasmonic coupling, resulting in less noisy spectra.^[^ ^47^ ^]^ At the same time, the enhancement is localized in proximity of the tip apex and the spatial resolution reaches up to tens of nanometres.^[^ ^45^ ^]^ The improved resolution allows for a better correlation between Raman spectra and STM/AFM morphological features and a distinction between different types of defects.^[^ ^46^ ^]^ In addition to the previously cited techniques, there are other SPM methods utilized to investigate specific characteristics of defects. However, these methods are rarely employed and are not extensively discussed in this context. For more information, interested readers can refer to refs. [11, 12, 48, 49].

**Table 1:** Summary of the most employed characterization techniques for defects in WS_2_ MLs.

Technique	Provided information	Refs.
Transmission electron microscopy (TEM)	Identification of the defect site,	[34, 37]
	statistical quantification of defect density,	
	evaluation of the strain in the defect site	
X‐ray photoemission spectroscopy (XPS)	Identification of the chemical nature of defects,	[50, 51, 52]
	quantification of defect density,	
	evaluation of n/p doing action of defects	
Raman spectroscopy	Qualitative and quantitative evaluation of defect density,	[35, 53, 54]
	qualitative and quantitative evaluation of electron density,	
	identification of strain due to defects	
Field‐effect transistor (FET) characterization	Evaluation of n/p doing action of defects,	[34, 36]
	evaluation of the electron density,	
	evaluation of the transport property parameters	
Photo‐luminescence (PL) measurement	Study of different exciton species,	[35, 55, 56]
	quantification of carrier and defect density,	
	analysis of different recombination processes	
Scanning tunneling microscopy (STM)	Identification and classification of defects	[38, 39]
Scanning tunneling spectroscopy (STS)	Measurement of the LDOS of defects	[40, 41]
CO‐tip atomic force microscopy (AFM)	Identification of the defect site	[38, 44]
Tip‐enhanced Raman spectroscopy (TERS)	Same information of standard Raman analysis with an enhanced and spatially localized signal	[45, 46, 47]
Ab initio simulation	Prediction of band diagram and DOS of defects,	[38, 52, 57]
	evaluation of formation energies of defects,	
	prediction of the optical absorption/emission properties	

Finally, ab initio simulation techniques are a very powerful tool to predict the properties of defects in WS_2_ ML and validate experimental findings.^[^ ^38^, ^58^ ^]^ Through these simulations, it becomes feasible to anticipate the electronic characteristics of materials and defects by modeling the electronic band diagram and the density of states (DOS). This information is crucial, as the electronic and optical properties of a pristine semiconductor are connected to the behavior of free electrons and holes in the conduction and valence band, respectively.^[^ ^59^, ^60^ ^]^ This picture is altered by defects that can be responsible for additional states in the forbidden energy gap between the conduction and valence band. Defect states in the energy gap are easily recognizable since they appear as flat (i.e., dispersionless) in the band diagram.^[^ ^38^ ^]^ Occupied defect states are classified as donors since they can release electrons to the conduction band of the material, when they get ionized. On the contrary, acceptor states are empty defect states that acquire electrons from the valence band when ionized, and increase the hole concentration in the valence band.^[^ ^61^ ^]^ Notice that defect states can alter the free carrier concentration (doping effect) only if they are shallow, that is, the states are sufficiently close in energy to the valence or conduction band such that thermal energy is sufficient to activate the carrier transition between the defect states and the bands (ionization of the defect). Hence, the analysis of the defect states in the band diagram is useful to predict their donor/acceptor nature and understand if the defect is an effective dopant.^[^ ^61^ ^]^ Moreover, the band diagram also provides some guidance in the interpretation of the optical properties of WS_2_, since optical absorption/emission are associated to electronic transitions between the conduction and valence band. For example, shallow states are able to reduce the effective energy distance between occupied states in conduction band and empty states in valence band, decreasing the optical transition energy and causing a red‐shift in the absorption and emission spectra.^[^ ^62^, ^63^ ^]^ On the contrary, the presence of deep defect states in the band diagram is usually a marker of suppressed PL, since these states can act as trap levels able to mediate non‐radiative recombination, competing with radiative transitions.^[^ ^60^, ^64^ ^]^ Last, identifying effective acceptor or donor states within the band diagram offers insights into the expected changes in the relative stabilization of neutral exciton and negative trion populations resulting from variations in hole and electron concentrations (i.e., Fermi level shifts). Consequently, in numerous instances, the enhancement or suppression of PL and the shift in PL due to exciton‐trion conversion, as discussed in Section 2, can be anticipated through the analysis of defect states in the band diagram.^[^ ^17^, ^65^ ^]^

## 4 Intrinsic WS_2_ Defects

Intrinsic defects are unintentionally present in the MLs, forming during the sample preparation. Their origin and nature are strongly correlated to the adopted synthesis approach, so the intrinsic defect types can vary in the different works.^[^ ^38^ ^]^ Here we report the most common and relevant ones.

### 4.1 Sulfur Vacancies

Defects at the chalcogen site are the most reported intrinsic defects in TMDs as WS_2_.^[^ ^13^, ^39^ ^]^ These defects are commonly identified as sulfur mono‐vacancies (V_S_).^[^ ^21^ ^]^ Their presence is reported in all types of WS_2_ MLs, irrespective of the synthesis approach (i.e., mechanical exfoliation (ME) from bulk material,^[^ ^66^, ^67^ ^]^ chemical vapor deposition (CVD),^[^ ^12^, ^68^ ^]^ epitaxial growth^[^ ^69^ ^]^). Nevertheless, the densities of sulfur vacancies varies among the different synthesis procedures. It is well established that exfoliated samples are characterized by a lower defect density than other approaches,^[^ ^56^ ^]^ while the physical properties of CVD‐synthesized MLs are markedly limited by a high defect density.^[^ ^11^, ^64^ ^]^ The typical range of V_S_ density in CVD samples is quite wide, from 10^10^–10^11^ cm^−2^ up to 10^12^–10^13^ cm^−2^.^[^ ^11^, ^54^, ^70^ ^]^ In addition, the spatial distribution of sulfur vacancies is often non‐uniform within a ML: many experimental evidences show that the sample edges (above all in the CVD ones) are characterized by a higher defect density than the central areas of the ML.^[^ ^12^, ^71^, ^72^ ^]^ Other works reveal that the vacancy concentration can be high also in the middle of the ML in correspondence of the nucleation center^[^ ^11^ ^]^ or in the threefold domains in hexagonal‐shape samples.^[^ ^73^ ^]^ The variability of V_S_ concentration is ascribed to the different synthesis recipes, but can also be associated to post‐growth factors. For example, an in‐vacuum annealing at about 600 °C is responsible for an increase in the vacancy density, but also intense electron beams can create new vacancies during electron microscopy characterizations.^[^ ^40^ ^]^ Finally, the ambient atmosphere oxidation also reduces the V_S_ density by oxygen passivation.^[^ ^74^ ^]^ For that reason, to better clarify the abundance of sulfur vacancies in the different conditions, several theoretical efforts have been performed to evaluate the formation energy of V_S_, trying to consider the synthesis parameters^[^ ^38^ ^]^ and include all possible types of intrinsic defects that might compete with the V_S_ formation.^[^ ^40^, ^57^ ^]^ Ab initio simulations show that sulfur mono‐vacancies have the lowest formation energy among other intrinsic defects that do not involve hetero‐atoms,^[^ ^57^ ^]^ hence V_S_ is expected to be the most common defects in WS_2_ MLs. However, in the presence of molecules or contaminants (e.g., oxygen species), other types of defects can compete and/or passivate the sulfur vacancies. For example, it is proven that in “as‐grown” CVD MLs, the V_S_ concentration is low due to the abundance of passivating oxygen substituent atoms and the vacancies are mainly created in the post‐growth steps.^[^ ^40^, ^41^ ^]^

**Figure 2:**
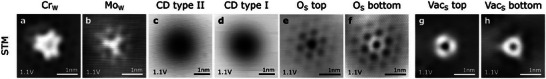
STM topographies of different defects observed in “as‐grown” CVD‐WS_2_: a) Cr_W_, b) Mo_W_, c,d) different types of charged defects CDs, e,f) O_S_ in the top/bottom S plane, g,h) S vacancies Vac_S_ in the top/bottom *S* plane. Reproduced with permission.^[^ ^38^ ^]^ Copyright 2019, American Chemical Society.

The sulfur vacancy location is usually identified through TEM analysis, since the signal intensity from the V_S_ site is generally lower than that of the nearest six S atoms.^[^ ^72^, ^75^ ^]^ Nevertheless, this approach leads to an ambiguous distinction between V_S_ and other sulfur substituent (e.g., O_S_).^[^ ^41^ ^]^ So, STM is proposed as an approach to uniquely identify the V_S_ sites.^[^ ^38^, ^40^ ^]^ STM mappings of different intrinsic defects reported in **Figure** **2** clearly show that V_S_ can be easily distinguished from other defects due to a unique electronic pattern.^[^ ^40^ ^]^

#### 4.1.1 Electronic and Transport Properties

**Figure 3:**
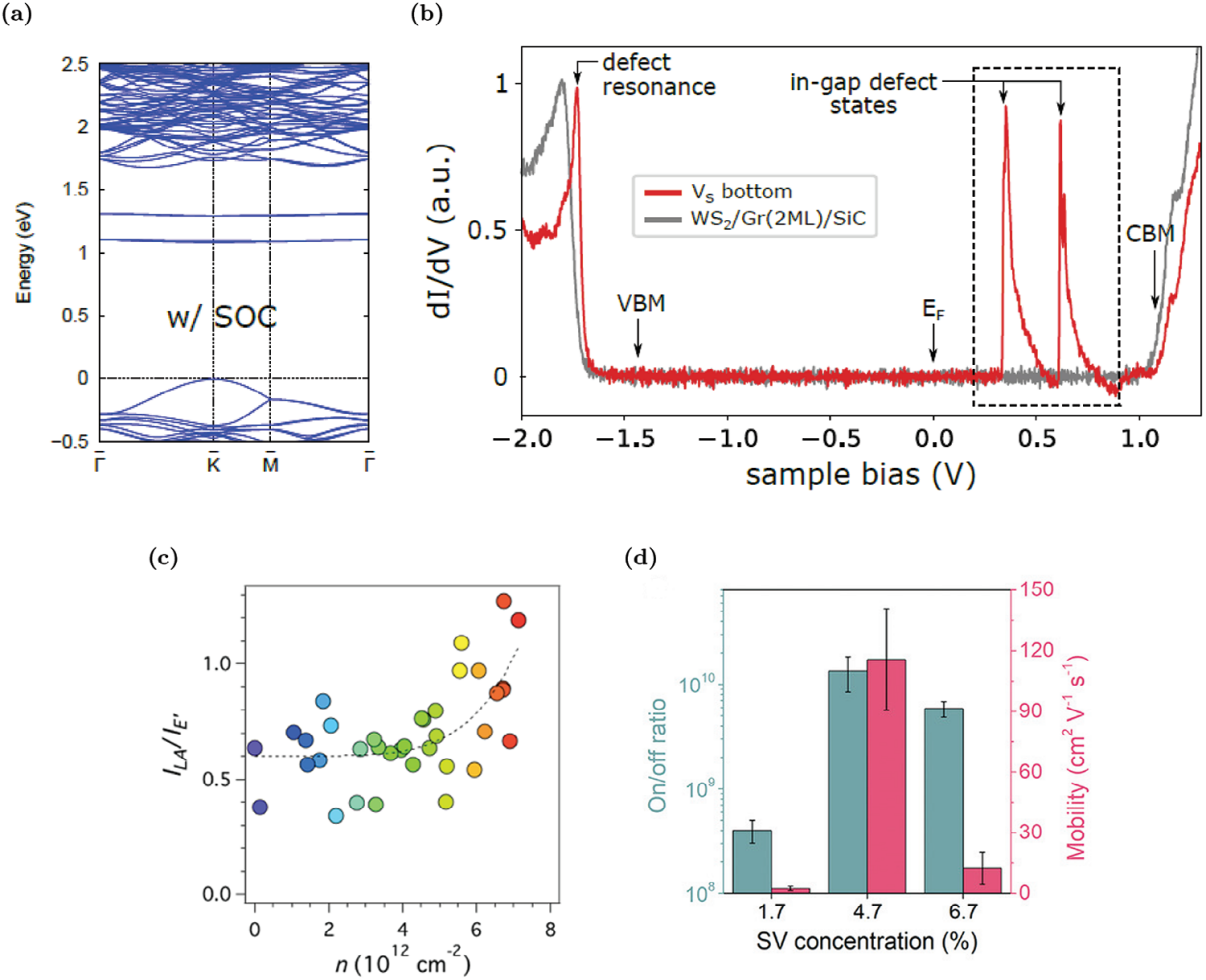
Electronic and transport properties of WS_2_ MLs with sulfur vacancies: a) sulfur vacancy band diagram, calculated at DFT level considering spin‐orbit coupling (SOC). Reproduced with permission.^[^ ^38^ ^]^ Copyright 2019, American Chemical Society. b) STS spectra recorded on a V_S_ and in the pristine WS_2_. Reproduced with permission.^[^ ^40^ ^]^ Copyright 2019, American Physical Society. c) Correlation between defect density and electron concentration: the ratio between the intensity of the *LA* and *E*′ Raman peaks (i.e., $I_{LA}/I_{E^{\prime }}$) is a common parameter to monitor the variation of the V_S_ density. Reproduced with permission.^[^ ^54^ ^]^ Copyright 2019, IOP Publishing. d) On/off current ratios and field‐effect mobilities of FETs with different S vacancy (SV) concentrations. Reproduced with permission.^[^ ^75^ ^]^ Copyright 2021, Wiley‐VCH.

The main electronic fingerprint of a sulfur mono‐vacancy is the presence of in‐gap states, which form two pairs of nearly‐degenerate flat bands in the gap, as proven by calculated band diagrams (**Figure** **3**a). A detailed analysis of the contributions of the different chemical species reveals that W 5d orbitals and, minimally S 3p states are the major contributions to the in‐gap states.^[^ ^40^ ^]^ The contribution of d orbitals of a heavy element, W, is responsible for the large splitting between the in‐gap states (about 250 meV) due to spin‐orbit coupling (SOC).^[^ ^40^ ^]^ Additional occupied defect resonant states (i.e., energetically degenerate with the pristine bands) are hardly visible slightly below the valence band maximum (VBM).^[^ ^40^ ^]^ The simulated electronic picture is completely in agreement with the experimental evidence from STS spectra (Figure 3b).^[^ ^40^ ^]^ As discussed in Section 3, the measurement of the differential conductance above the defect site in the STS set‐up, provides direct access to the DOS of the defect and proves the existence of the double in‐gap states as well as of the resonant peak in the valence band. Simulations and experimental measurements also agree with the evidence that the in‐gap states are empty (i.e., above the Fermi level) and quite deep.^[^ ^40^ ^]^ According to STS spectra, the in‐gap states are 774 ± 5 and 522 ± 5 meV below the conduction band minimum (CBM).^[^ ^40^ ^]^ These features are important in the discussion about the donor/acceptor nature of V_S_. A more detailed analysis of the STS spectrum shows an energy broadening and satellite peaks close to defect states that are not reproduced in the simulation: these effects are due to tunneling of electrons inelastically scattered by phonons.^[^ ^40^ ^]^

The effects of the defect states associated with sulfur vacancies on the electronic properties of WS_2_ have been controversially discussed for a long time, attributing to V_S_ the role of both donor and acceptor defects.^[^ ^76^ ^]^ Different works claim that the vacancies are associated with donor effects.^[^ ^73^, ^77^ ^]^ This thesis is supported by experimental evidence that detects a positive correlation between electron concentration and V_S_ density in TMDs as MoS_2_ and WS_2_.^[^ ^53^, ^54^, ^75^ ^]^ For example, Figure 3c shows that the electron concentration clearly increases together with the Raman peak *I*_*LA*_ intensity which is sensitive to defects.^[^ ^54^ ^]^ Hence, the sulfur vacancy was commonly considered the origin of the intrinsic n‐type nature of WS_2_.^[^ ^55^, ^77^ ^]^ However, recently, this thesis has been strongly rejected. Ab initio analysis points out that in the isolated ML the vacancy states are unoccupied, so they are not able alone to promote electrons to the conduction band.^[^ ^57^ ^]^ On the contrary, V_S_ is a deep and ineffective acceptor, and it cannot be the origin of the intrinsic n‐doping in WS_2_.^[^ ^57^ ^]^ A solution for the contrast between experimental evidence and theoretical simulations is proposed in different works.^[^ ^54^, ^75^ ^]^ It is supposed that the substrate below the TMD samples is responsible for a charge transfer able to alter the carrier concentrations of the ML. For example, hydrogenated defects on the surface of SiO_2_ (the most commonly employed substrate for TMDs) can ionize and provide additional electrons to the WS_2_ MLs.^[^ ^78^ ^]^ Hence, the n‐type nature of WS_2_ MLs might be associated to the charge transfer from the substrate.^[^ ^78^ ^]^ The carrier modulation in WS_2_ due to the substrate is also highlighted by different experimental works that analyze the variation of ML properties on different substrates as well as self‐standing.^[^ ^79^, ^80^ ^]^ In this framework, electrons from the substrate can be injected in the WS_2_ ML and populate the in‐gap states of the sulfur vacancy.^[^ ^54^, ^75^ ^]^ Hence, the vacancy states, populated by the substrate, behave as donor levels and can modify the carrier concentration in the conduction band. The thesis that in‐gap states can be populated is confirmed by theoretical evaluations^[^ ^72^ ^]^ and experimental observations^[^ ^39^ ^]^ that identify the presence of negatively charged sulfur vacancies in n‐type WS_2_. In short, according to this interpretation, sulfur vacancies are not the direct cause of the n‐type nature of WS_2_, but they are a channel that facilitates the electron transfer from the substrate to the conduction band of the ML.^[^ ^54^ ^]^ However, this interpretation has not been completely accepted yet, and there are other works that try to associate the n‐doping nature of WS_2_ to other intrinsic defects (e.g., H impurities as discussed in Section 4.4) and not to V_S_.

In addition to carrier modulation, the sulfur vacancies also influence the electron mobility. Differently from the common notion of mobility degradation due to defects, it is observed that sulfur vacancies can enhance the mobility.^[^ ^73^, ^75^ ^]^ The origin of this effect is not uniquely identified: it is proposed that the lattice elongation due to the atom relaxation in correspondence with the vacancy site can be responsible for a variation of the effective mass and a subsequent mobility improvement.^[^ ^68^, ^73^ ^]^ Other works state that the sulfur vacancies promote the electron mobility boost via hopping‐transport and not in band‐transport.^[^ ^75^ ^]^ According to the latter thesis, electron transport occurs due to hopping from a localized vacancy state to another one and the vacancy concentration is a key parameter in order to control the hopping probability between the localized sites. As reported in Figure 3d where the performance of FETs with different V_S_ concentrations is summarized, the maximum mobility improvement is achieved in cases of intermediate defect densities. Low densities result in a too‐large spatial separation between hopping sites, while large concentrations induce a vacancy clustering tendency that alters the positions of the defect levels in the gap and increases the activation barrier for hopping processes.^[^ ^75^ ^]^

#### 4.1.2 Optical Properties

**Figure 4:**
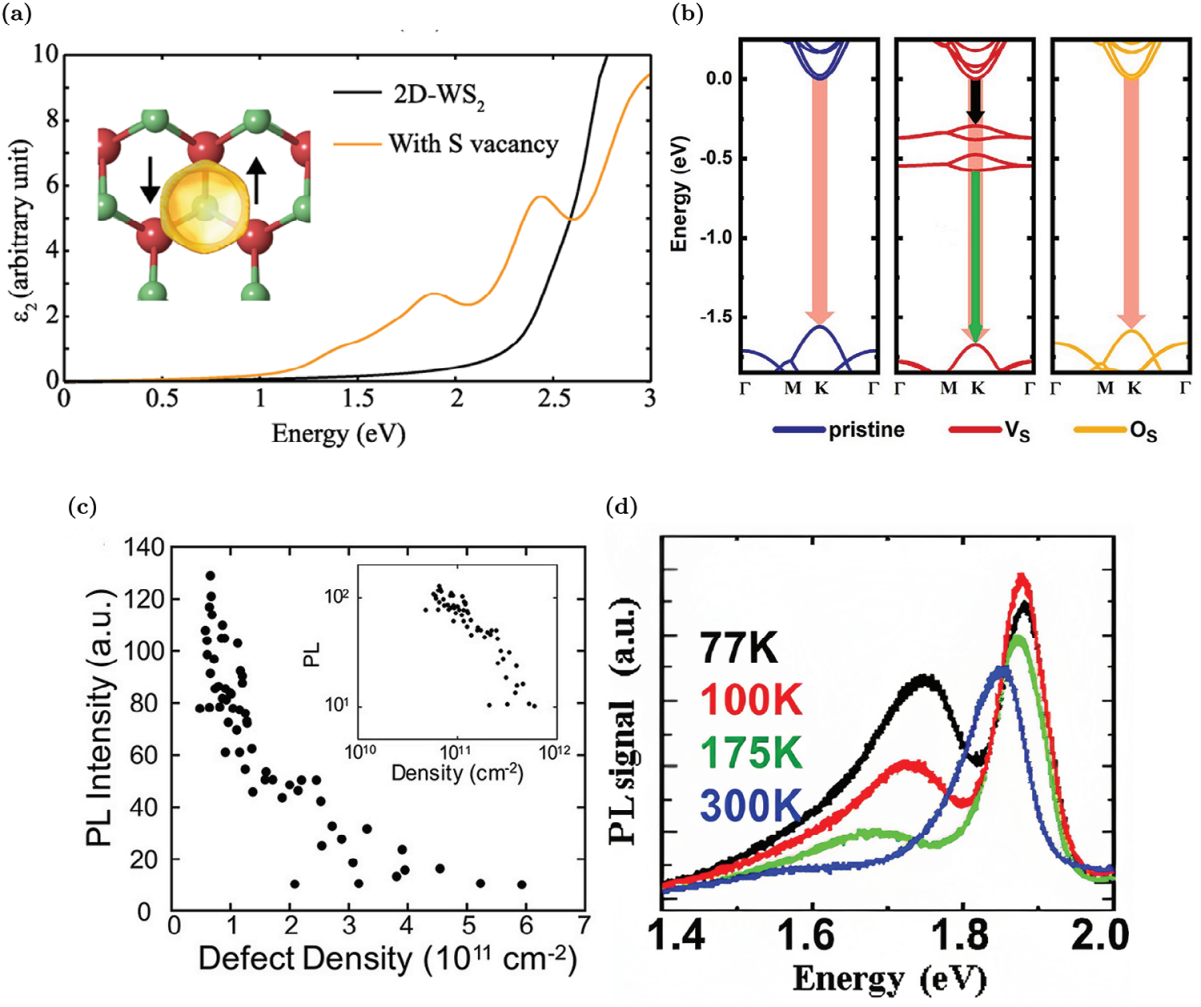
Optical properties of WS_2_ MLs with sulfur vacancies: a) Imaginary part of the dielectric function of V_S_‐WS_2_ (yellow) and pristine WS_2_ (black), respectively. The inset shows the polarized spin density of V_S_. Reproduced with permission.^[^ ^57^ ^]^ Copyright 2021, American Physical Society. b) Schematic representation of the different carrier transitions in the pristine WS_2_, S vacancy, and O substituent case: pale‐red, green, and black arrows represent band‐to‐band radiative, defect‐bound exciton radiative and trap assisted non‐radiative recombinations, respectively. Adapted with permission.^[^ ^60^ ^]^ Copyright 2021, American Chemical Society. c) Correlation between PL intensity and V_S_ density. Reproduced with permission.^[^ ^11^ ^]^ Copyright 2018, American Chemical Society. d) PL spectra measured over the temperature range from 77 to 300 K of a WS_2_ with the V_S_‐induced PL peak. Reproduced with permission.^[^ ^56^ ^]^ Copyright 2022, American Chemical Society.

Considering the presence of additional states in the gap due to V_S_, the optical properties of V_S_‐rich WS_2_ MLs are also modified. For example, the optical transitions mediated by the vacancy states alter the absorption spectrum of WS_2_: the simulated imaginary part of the dielectric function, reported in **Figure** **4**a, shows an additional peak at energy lower than the optical gap of the pristine material.^[^ ^57^, ^72^, ^81^ ^]^ The absorption edge of WS_2_ ML is red‐shifted from about 2 eV in the pristine material to about 1.4 ÷ 1.5 eV (infrared region) in the V_S_‐rich samples.^[^ ^57^, ^72^ ^]^

The emission properties of the material are also changed by the presence of the vacancy induced defect states. The latter act as deep levels that can trap carriers from the conduction band.^[^ ^56^, ^60^ ^]^ Hence, as depicted in Figure 4b, the conduction band electrons can decay in a non‐radiative way in the V_S_ states, contrasting the band‐to‐band radiative recombination that is the predominant mechanism in the pristine samples.^[^ ^60^ ^]^

The negative effects of V_S_ on the emission properties are confirmed experimentally by the drop of the radiative lifetime (more than one order of magnitude decrease) together with the increase of the non‐radiative processes mediated by defect states as the vacancy concentration rises.^[^ ^56^ ^]^ The evident result of the V_S_ role is a significant suppression of the PL emission (also larger than one order of magnitude), as shown in Figure 4c, where an inverse correlation between PL intensity e V_S_ concentration is clearly detected.^[^ ^11^, ^56^, ^60^ ^]^ In addition to the PL suppression as the V_S_ density increases, the emission peak also tends to slightly red‐shift due to a major suppression of the exciton component with respect to the trion one.^[^ ^12^, ^56^, ^60^ ^]^ As discussed in Section 2, this exciton‐to‐trion conversion as the V_S_ density rises, is compatible with the increase in the electron concentration. Consequently, this red‐shift is considered as proof of the electron concentration increase in V_S_‐rich regions.^[^ ^54^ ^]^

Considering the negative effects of V_S_ on the emission properties (the internal quantum efficiency of V_S_‐rich WS_2_ MLs can drop below 1%^[^ ^56^ ^]^), different approaches have been proposed to passivate the sulfur vacancies and improve the optoelectronic properties of WS_2_. These strategies, which mostly rely on chalcogen substituent atoms as well as molecule chemisorption, are more extensively discussed in the next sections. Despite the suppression of the main PL peak (i.e., neutral exciton and negative trion components), sulfur vacancies can be employed to enhance new emission peaks associated to the defect‐bound excitons.^[^ ^56^, ^72^ ^]^ As discussed in Section 2, at cryogenic temperatures, carriers trapped in the vacancy states can radiative recombine from a defect bound‐exciton associated to the vacancy states, as schematized in Figure 4b (green arrow).^[^ ^72^ ^]^ The vacancy‐bound emission peak is at about 1.7 ÷ 1.8 eV,^[^ ^12^, ^56^, ^72^ ^]^ depending on the specific sample characteristics and measurement temperature. Hence, this peak is well below and isolated from the main emission peak and it can become the predominant one at low temperatures, as shown in Figure 4d.^[^ ^56^, ^72^ ^]^ The localization of this bound‐exciton and the sharp spectral separation of this emission peak make the V_S_ defect appealing for single‐photon emitters (SPEs).^[^ ^57^ ^]^ The single‐photon emission from V_S_ was experimentally achieved, employing intrinsic^[^ ^48^ ^]^ as well as intentionally generated sulfur vacancies.^[^ ^82^ ^]^ V_S_ is also able to enhance SHG, up to two orders of magnitude with respect to the pristine material.^[^ ^32^ ^]^ This is possible because the in‐gap defect states improve the adsorption of coupled photons, at energy smaller than the energy gap, that can be converted in a photon at doubled energy.

### 4.2 Oxygen Substituents

**Figure 5:**
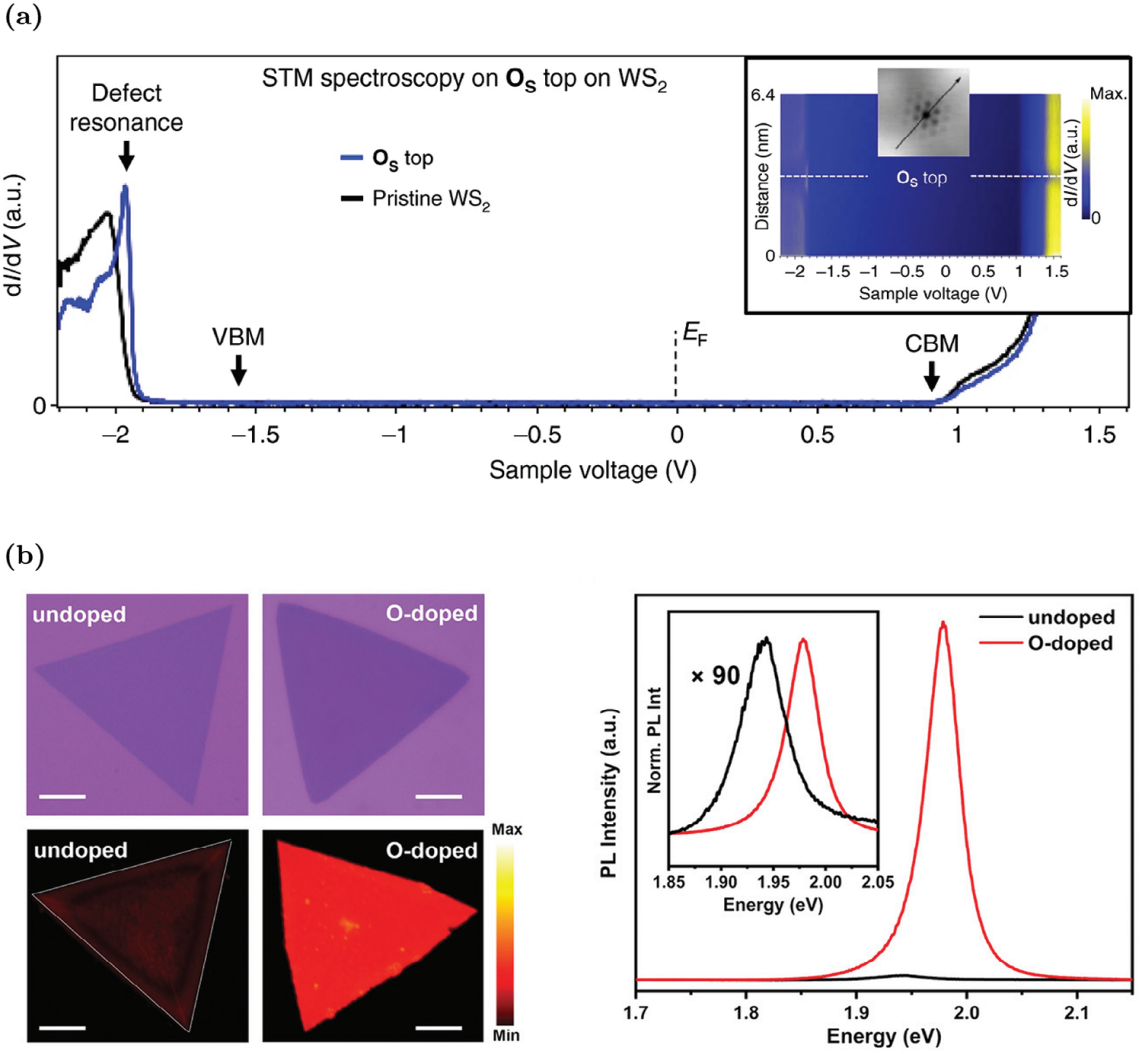
Properties of WS_2_ MLs with oxygen substituent defects: a) STS spectra acquired on an O_S_ site (blue line) and in the pristine (black line) WS_2_, the voltage of 0 V corresponds to the Fermi level. Reproduced under the terms of the CC‐BY 4.0 license.^[^ ^41^ ^]^ Copyright 2019, The Authors, Published by Springer Nature. b) (Left) Optical images and the corresponding integrated PL intensity mappings of O‐doped and undoped WS_2_ (scale bars: 20 μm). (Right) Associated PL spectra at 293 K (inset shows normalized spectra). Reproduced with permission.^[^ ^64^ ^]^ Copyright 2021, Wiley‐VCH.

Although sulfur vacancies are considered the most abundant intrinsic defects in the WS_2_ MLs, more accurate experimental investigations reveal an extensive presence of oxygen atoms replacing the sulfur ones (O_S_) in “ as‐grown” samples, above all in the case of CVD synthesis.^[^ ^41^ ^]^ Indeed, O_S_ is more thermodyamically favorable than the sulfur vacancy^[^ ^38^, ^40^ ^]^ and oxygen atoms can be easily incorporated during the CVD synthesis in the presence of O sources (e.g., the WO_3_ precursor), during mild annealing in an oxygen atmosphere or simply in prolonged exposure to the atmospheric environment.^[^ ^41^ ^]^ The density of intrinsic O_S_ defects is frequently underestimated compared to V_S_, primarily due to the experimental complexities involved in distinguishing between these two defects.^[^ ^41^ ^]^ TEM characterizations are not effective at distinguishing O_S_ from V_S_ due to the poor contrast associated with the low atomic number of oxygen.^[^ ^64^ ^]^ The true atomic resolution AFM (CO‐tip AFM) technique is also not effective, since the oxygen substituent atoms are located slightly below the surface sulfur plane and can be confused with vacancies.^[^ ^41^ ^]^ For that reason STS/STM measurements are proposed as a method to unequivocally identify O_S_.^[^ ^41^ ^]^ The effectiveness of STM patterns in distinguishing between the O_S_ and V_S_ is shown in Figure 2. Differently from the in‐gap states of V_S_, the O_S_ band diagram is characterized by the absence of defect states in the gap region, as proven by theoretical simulations (Figure 4b)^[^ ^38^ ^]^ and experimental STS (**Figure** **5**a).^[^ ^41^ ^]^ The energy gap is also unaffected by O_S_ presence. The unique feature associated with O_S_ is a resonant defect state inside the valence band that minimally alters the material properties.^[^ ^41^ ^]^ The similarity between O_S_ and the pristine band diagram is associated to the isovalence nature of O and S species. So, the in‐gap states due to the dangling bonds of the V_S_ are perfectly saturated by the oxygen atom. Considering the negative effects of in‐gap states due to V_S_, the intentional incorporation of oxygen atoms in WS_2_ is considered as an effective approach to passivate the sulfur vacancies and improve the material properties.^[^ ^62^, ^64^ ^]^ Different strategies are adopted to incorporate oxygen atoms, such as CVD synthesis in which Fe_2_O_3_, as O source, is mixed with the W precursor before sulfurization,^[^ ^64^ ^]^ mild O^2+^ plasma treatment,^[^ ^56^ ^]^ O^+^ ion bombardment at low doses^[^ ^60^ ^]^ and laser‐assisted O_2_ chemisorption.^[^ ^53^, ^62^ ^]^ The latter approach is more extensively discussed in Section 5.4. O_S_ effects are usually discussed in view of the passivation of V_S_ that is always present in the samples. For example, MLs in which V_S_ is passivated by O are characterized by lower electron concentration with respect to V_S_‐rich samples with a markedly evident n‐type nature.^[^ ^64^ ^]^ Although O_S_ minimally alters the crystal quality, samples with O_S_ have lower electron mobility with respect to the V_S_‐rich MLs due to the role of sulfur vacancies in the hopping transport mechanism.^[^ ^64^ ^]^ However, O atoms are intentionally incorporated above all to enhance the optical properties of defective WS_2_ MLs. The passivation of the trap‐states associated to the sulfur vacancies suppresses the non‐radiative recombination processes mediated by the vacancy states and increases the radiative lifetime.^[^ ^56^, ^64^ ^]^ Hence, the evident result of the O_S_ incorporation is a significant enhancement of the PL emission.^[^ ^56^, ^60^, ^64^ ^]^ For example, the decrease of the V_S_ concentration from 4% to 1.5% and the rise of O_S_ density up to 3.5% are responsible for a 90‐fold PL increase (Figure 5b).^[^ ^64^ ^]^ Moreover, the peak slightly blue‐shifts (about 30 meV) and its full‐width‐half‐maximum (FWHM) decreases due to the trion‐to‐exciton conversion. ^[^ ^64^ ^]^ As discussed in Section 2, this is a consequence of the reduction of the electron concentration due to O_S_.

The passivation of V_S_ and the absence of in‐gap states associated with O_S_ is also appreciable in the low‐temperature PL spectra, where the defect‐bound exciton peak is almost suppressed.^[^ ^56^, ^64^ ^]^ Finally, it is important to notice that the properties of WS_2_ MLs in which the sulfur vacancies are passivated by O_S_ are less prone to deterioration due to sample aging.^[^ ^64^ ^]^ Indeed, it is known that WS_2_ tends to oxidize in ambient atmosphere and the sulfur vacancies accelerate the conversion of WS_2_ to its correspondent oxide.^[^ ^71^, ^74^ ^]^ Hence, the intentional O_S_ defect passivating the vacancies is believed to prevent further uncontrollable oxygen incorporation due to aging.

In conclusion, a limited concentration of oxygen atoms in WS_2_ has beneficial effects, above all on the optical properties. However, high densities of O_S_ are detrimental to the PL because the tungsten oxide, which originated from the oxidation of large areas of WS_2_, has poor PL capabilities.^[^ ^53^, ^62^ ^]^

### 4.3 Tungsten Vacancies and Other Intrinsic Defects

Differently from the extensively studied sulfur vacancies, tungsten vacancies (V_W_) are less investigated. Indeed, theoretical simulations reveal that the formation energy of this type of vacancy is significantly larger than the V_S_ one (i.e., about 1.5 and 6.5 eV for V_S_ and V_W_, respectively).^[^ ^57^ ^]^ Experimental characterizations of “as‐grown” samples confirm the presence of a few V_W_ defects.^[^ ^38^ ^]^ However, there are some cases in which the V_W_ presence is significant. For example, when WS_2_ MLs are synthesized as hexagonal flakes, three‐fold subdomains (commonly labeled as β regions) characterized by higher densities of V_W_ are present.^[^ ^68^, ^73^ ^]^ These areas are the result of the enlargement of triangle‐shaped flakes, under W‐poor growth conditions, near the corners of the triangle.^[^ ^73^ ^]^ In addition, it is possible to identify V_W_ or complexes of W vacancies surrounded by missing S atoms, in damaged samples (e.g., exposed to ion bombardments or prolonged laser treatments).^[^ ^60^ ^]^ Despite the limited presence of W vacancies, the few cases of samples with V_W_ show a large deterioration of their properties.

**Figure 6:**
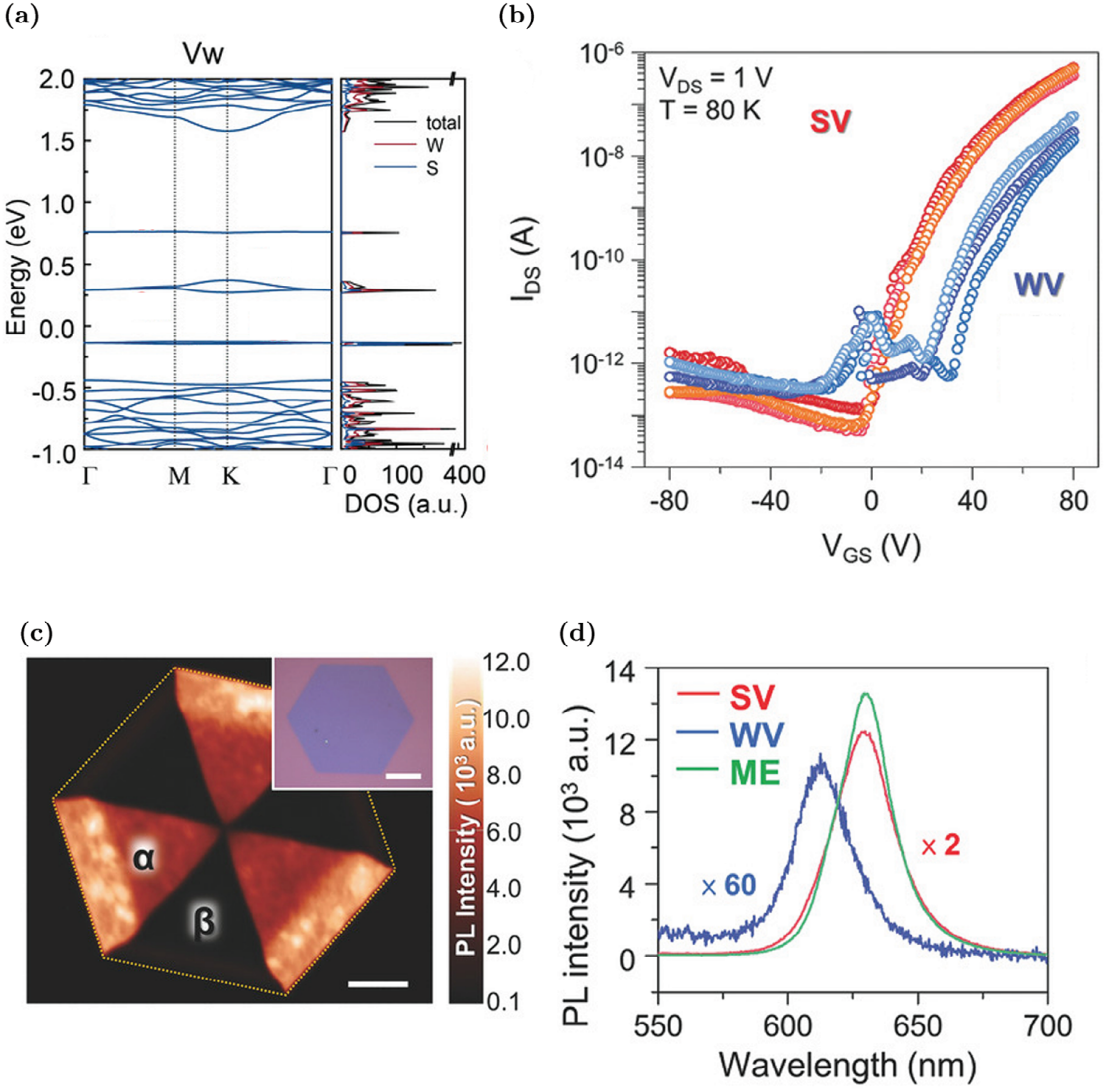
Properties of WS_2_ MLs with tungsten vacancies: a) Band structure and DOS for single V_W_. The Fermi level is set to be zero. Reproduced with permission.^[^ ^83^ ^]^ Copyright 2022, Wiley‐VCH. b) Comparison of the transcharacteristics of FET devices realized in V_S_‐rich (SV) and V_W_‐rich (WV) domains. Reproduced with permission.^[^ ^73^ ^]^ Copyright 2017, Wiley‐VCH. c) PL intensity mapping image of a hexagonal WS_2_ ML with contrasts between α and β domains. Inset shows the optical image of the sample, scale bars 10 μm. Reproduced with permission.^[^ ^73^ ^]^ Copyright 2017, Wiley‐VCH. d) Comparison of the PL spectra of a mechanically exfoliated (ME) sample with low defect density, V_S_‐rich (SV) and V_W_‐rich (WV) domains in a CVD sample. Reproduced with permission.^[^ ^73^ ^]^ Copyright 2017, Wiley‐VCH.

Theoretical simulations have shown that the dangling bonds associated to the sulfur atoms surrounding the V_W_ site are responsible for the formation of different in‐gap states. Specifically, there is a group of occupied defect states (mainly due to the p orbitals of S) and two groups of empty ones (mainly due to the d orbitals of W), as visible in **Figure** **6**a.^[^ ^38^, ^57^, ^81^ ^]^ The latter defect states are supposed to behave as acceptor levels,^[^ ^77^ ^]^ since W vacancies can exist in a neutral or in a negatively charged configurations (i.e., ionized acceptor).^[^ ^57^ ^]^ This feature is confirmed by electrical‐transport characterizations that reveal a decrease in the conduction band electron concentration when V_W_ is present.^[^ ^73^ ^]^ By comparing the transcharacteristics of FETs fabricated with V_S_‐rich and V_W_ domains (Figure 6b), it becomes apparent that V_W_ defects are responsible for an up‐shift of the threshold voltage. This shift is attributed to a decrease in the intrinsic electron concentration. In addition, the carrier mobility is reduced due to the deep trap states in V_W_‐rich regions.^[^ ^73^ ^]^ However, the most detrimental effects of V_W_ are visible in its optical properties. The V_W_ states are deep trap levels that enhance non‐radiative recombinations.^[^ ^60^ ^]^ The direct consequence is a strong PL suppression (emission drop can be larger than one order of magnitude), as visible in the PL map of a hexagonal ML where the V_W_‐rich domain (β domain) is highlighted (Figure 6c).^[^ ^60^, ^73^ ^]^ Notice that the PL quenching due to V_W_ is even more severe than the PL suppression due to V_S_, as shown in the PL spectrum in Figure 6d where the emission of low V_S_ concentration samples, V_S_‐rich and V_W_‐rich domains are compared.^[^ ^62^ ^]^ The V_W_ presence is also responsible for a blue‐shift of the emission peak due to the compressive strain associated to the V_W_ site or due to the trion‐to‐exciton conversion as consequence of the decrease in the electron concentration due to V_W_.^[^ ^73^ ^]^

Due to the detrimental effects of the W vacancies, these defects must be passivated. For example, it is proposed to add an erbium source during the synthesis to achieve Er substitutional doping, able to occupy the V_W_ sites.^[^ ^62^ ^]^ Er is selected for its atomic radius similar to W, enabling it to passivate V_W_ defects without creating additional deep trap states. This approach allows for a sixfold PL enhancement, with only a 7.9 nm red‐shift of the peak position due to shallow states associated to Er contribution.

In addition to V_S_ and V_W_, other intrinsic defects can be present in WS_2_ MLs, such as complexes of S and W missing atoms, di‐sulfur vacancies, interstitial and antisite defects involving S and W atoms.^[^ ^57^, ^83^ ^]^ In general, these defects are responsible for the creation of a series of in‐gap states that can act as deep donor or deep acceptor levels.^[^ ^57^ ^]^ However, their formation energy is very high and they are rarely observed.^[^ ^57^ ^]^ For that reason these intrinsic types of defects are not considered to be responsible for the spontaneous n‐type nature or any other major effect or physical property change in WS_2_ MLs.^[^ ^57^ ^]^

### 4.4 Hydrogen Impurities

**Figure 7:**
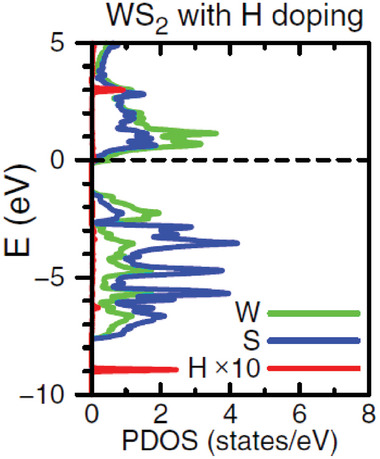
Projected density of states (PDOS) of a WS_2_ ML with a hydrogen atom at the hollow site. The Fermi level is set to zero and marked with a horizontal dashed line. Reproduced with permission.^[^ ^85^ ^]^ Copyright 2019, American Physical Society.

Considering the lack of consensus in the literature regarding the role of V_S_ defects as causes of the n‐doping of WS_2_, several works explore different unintentional impurity defects to identify possible donor species. Since hydrogen is always present in almost all growth environments (e.g., in H_2_S as S precursor for CVD WS_2_ samples^[^ ^38^ ^]^), it is highly likely to find H impurities incorporated in the basal plane^[^ ^39^ ^]^ or at the edges^[^ ^84^ ^]^ of the ML. From the theoretical analysis of the different sites in which H atoms can be incorporated, two preferential configurations are identified: H as an interstitial atom in the center of the hexagonal hollow site and H adatom over the S atom.^[^ ^57^, ^85^ ^]^ These two configurations have comparable formation energy^[^ ^57^ ^]^ and both cause minimal lattice distortion. Moreover, both configurations are responsible for an n‐doping effect.^[^ ^57^ ^]^ In the interstitial case, the H presence is able to raise the Fermi level at energies higher than the CBM, turning WS_2_ into an n‐type degenerate semiconductor, as shown in the DOS reported in **Figure** **7**.^[^ ^57^ ^]^ A noticeable increase in the electron concentration in the conduction band is achieved without creating any in‐gap states, so minimal thermal activation is required to ionize this impurity.^[^ ^85^ ^]^ On the contrary, when H atom is adsorbed over the S site, in‐gap states are generated slightly below the CBM.^[^ ^57^ ^]^ These levels are partially occupied and behave as donor states.^[^ ^57^ ^]^ Notice that the doping effect is less effective in the adatom configuration with respect to the interstitial one, due to the ionization energy required to promote electrons from the in‐gap states to the conduction band.^[^ ^85^ ^]^ However, theoretical simulations prove that both types of defects can exist as positive charged defects over a wide range of energies.^[^ ^57^ ^]^ For that reason, to challenge the role of V_S_ in the n‐doping of WS_2_, some studies propose H impurities as the origin of the intrinsic n‐type nature of the MLs.^[^ ^57^ ^]^ This thesis is confirmed by the experimental identification of positive charged defects that can be attributable to H impurities.^[^ ^39^ ^]^ In addition, some experimental works intentionally introduce H atoms in TMD MLs. For example, a study on different TMDs as MoTe_2_, WSe_2_ and MoS_2_, showed that intentionally H‐doped samples are characterized by a band‐transport conduction mechanism, while conduction by hopping mainly occurs in the pristine case.^[^ ^86^ ^]^ This proves that H‐doping is able to effectively push the chemical potential inside the conduction band. However, to the best of our knowledge, similar experimental characterizations have not been performed for WS_2_ yet.

In addition to the H atom, other foreign impurities can be unintentionally present in the WS_2_ MLs. For example, sodium atoms are supposed to be present in the synthesis environment and they can be adsorbed as adatoms on WS_2_ MLs.^[^ ^87^, ^88^ ^]^ The effects of Na adatoms will be thoroughly examined in Section 5.3, where surface charge transfer mechanisms by adatoms are discussed.

## 5 Intentional WS_2_ Doping

Intentionally introduced dopants are the most common approach to tune the electronic and optical properties of traditional semiconductors as well as of 2D semiconductors such as WS_2_. The most common doping approach is by atom substitution at the W as well as at the S site. Notice that substitutional atoms are not only intentionally introduced during or after the synthesis, but they also can be present in “as‐grown” samples due to contaminations during the synthesis (e.g., O_S_, Mo_W_, and Cr_W_^[^ ^38^ ^]^). Despite the widespread use of substitutional doping in conventional semiconductor technology, this could lead to ineffective carrier modulation in 2D materials due to the high ionization energies of dopant levels. The reduced electrostatic screening of 2D systems makes them more likely to have deep donor or acceptor levels that are unable to get ionized at common working temperatures.^[^ ^89^ ^]^ This means that the doping concentration in 2D materials must be higher (>1%) than in bulk semiconductors (typically at the part‐per‐million and part‐per‐billion levels) to be effective.^[^ ^20^ ^]^ For that reason, new and more effective doping approaches, such as surface charge transfer doping (SCTD), are employed.^[^ ^34^ ^]^ The large surface‐to‐volume ratio of MLs is responsible for the enhanced sensitivity toward adsorption. Hence, adatoms or adsorbed molecules are able to exchange carriers with the MLs and effectively tune the WS_2_ properties. Moreover, SCTD does not introduce significant distortion in the crystal structure of the underlying semiconductors.^[^ ^90^ ^]^ Nevertheless, this doping strategy is often considered insufficiently stable since adatoms/molecules can be easily desorbed and the doping effect is lost.^[^ ^91^, ^92^, ^93^ ^]^ Notice that both doping strategies (substitutional and SCTD) do not only aim at modulating the carrier concentrations, but also at passivating the detrimental intrinsic vacancies^[^ ^62^, ^64^, ^94^ ^]^ and adding new functionalities (e.g., single‐photon emission capability,^[^ ^95^ ^]^ magnetism,^[^ ^96^ ^]^ catalytic properties^[^ ^97^ ^]^) to the MLs.

A large number of W and S substitutional atoms, as well as adatoms and molecules, has been investigated by means of ab initio simulations,^[^ ^98^, ^99^ ^]^ however only a limited number of cases have also been experimentally characterized. In the next sections, the analysis mainly focuses on the doping approaches that have been investigated both theoretically and experimentally, as a more complete understanding of the effects of these doping strategies is available.

### 5.1 Tungsten Substituent Impurities

W atoms can be replaced by different types of metal species that are generally classified in three main categories: isoelectronic, n‐type, and p‐type substituents.^[^ ^20^ ^]^

#### 5.1.1 Isoelectronic W Substituents

**Figure 8:**
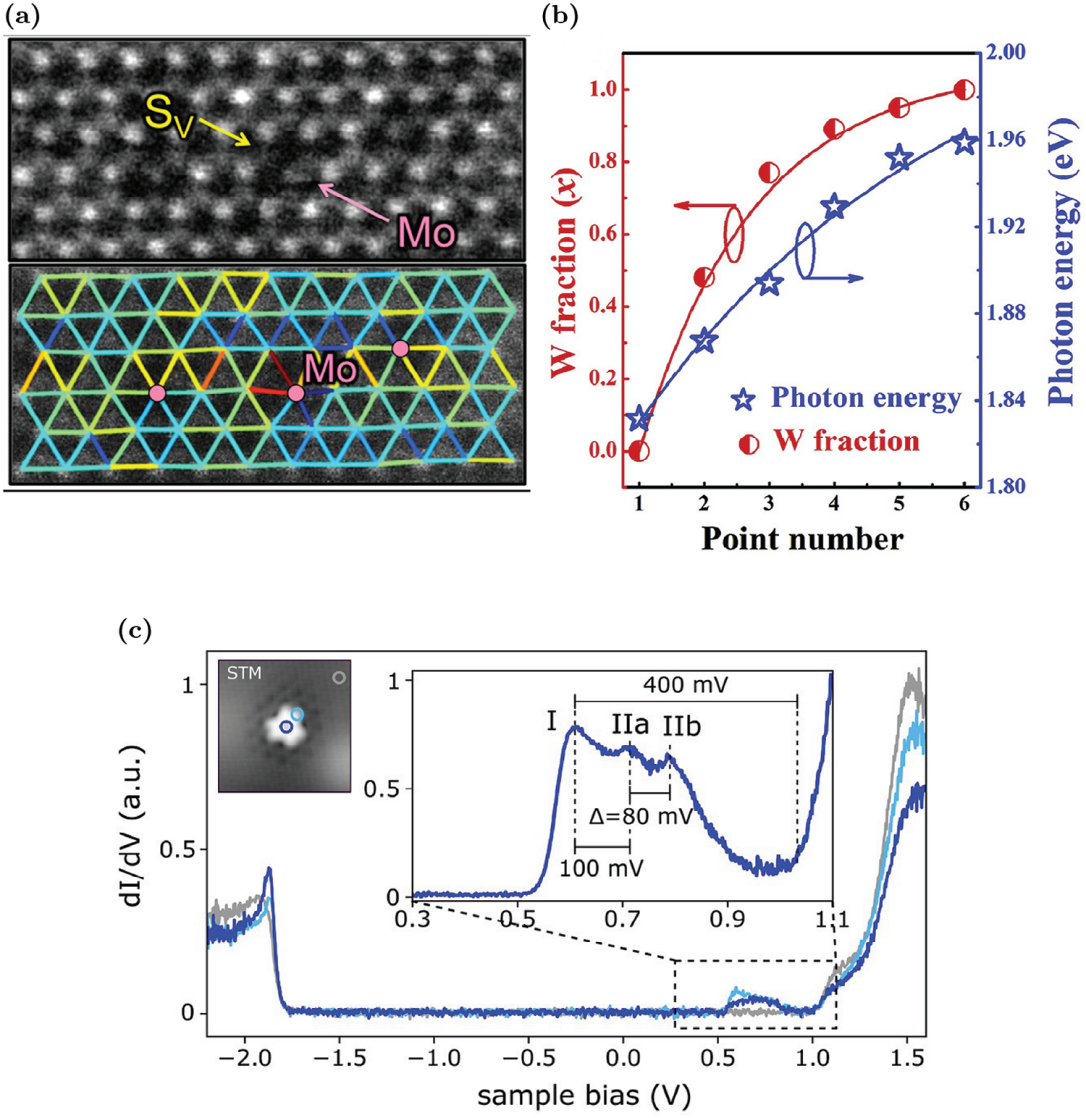
Effects of isoelectronic W substituent defects in WS_2_ MLs: a) ADF‐STEM image of WS_2_ with a Mo_W_ defect next to a S vacancy (S_V_) and measured displacement map of the same sample region. Reproduced with permission.^[^ ^101^ ^]^ Copyright 2017, American Chemical Society. b) Modulation of the W fraction and photon emission energy along the sample plane in a Mo_1−*x*_W_*x*_S_2_ ML. Reproduced with permission.^[^ ^102^ ^]^ Copyright 2019, Elsevier. c) STS measurements of a Cr_W_ defect. The circles in the inset image indicate the recorded spectra positions. Reproduced with permission.^[^ ^38^ ^]^ Copyright 2019, American Chemical Society.

Among isoelectronic substituents, molybdenum can replace W in WS_2_ MLs. Mo impurities are found in “as‐grown” CVD samples since Mo contamination can be present in the WO_3_ powder, commonly used as a W precursor.^[^ ^38^ ^]^ Theoretical evaluations confirm that Mo_W_ is thermodynamically favorable, especially in the W‐poor growth condition limit.^[^ ^38^, ^100^ ^]^ The similar atomic radius of W and Mo avoids any lattice distortion in correspondence with the Mo_W_ site.^[^ ^38^, ^101^ ^]^ The absence of strain and the similar electronic properties of W and Mo species result in the similarity between the properties of Mo_W_ and the pristine material since no defect states associated to Mo_W_ are created in the gap.^[^ ^38^, ^100^ ^]^ However, the Mo_W_ presence can have an influence on some WS_2_ sample features. It is observed the tendency of sulfur vacancies cluster in correspondence with Mo_W_ sites (about 80% of V_S_ in the analyzed samples are identified in correspondence of Mo_W_ defects).^[^ ^101^ ^]^ The co‐presence of Mo_W_ and V_S_ is shown by anular dark field STEM (ADF‐STEM; **Figure** **8**a). It has been proven that Mo_W_ favors the formation of negatively charged sulfur vacancies in its proximity and the complex Mo_W_‐V_S_ causes an evident lattice distortion, as shown in Figure 8a.^[^ ^101^ ^]^ This tendency to cluster can be exploited at the device fabrication level by intentionally introducing Mo atoms far away from the key functional areas of the device and employing Mo_W_ as “vacancy collector” to attract chalcogen vacancies to locations where they do not degrade the device properties.^[^ ^101^ ^]^ In addition to isolated Mo_W_ defects, the compatibility of W and Mo atoms is employed in the synthesis of Mo_1−*x*_W_*x*_S_2_ alloys.^[^ ^102^, ^103^ ^]^ The potentiality of this approach is confirmed by the CVD synthesis of a Mo_1−*x*_W_*x*_S_2_ alloy with a modulation of the in‐plane composition and a tunable PL peak position, from 1.83 eV (pure MoS_2_) to 1.96 eV (pure WS_S_).^[^ ^102^ ^]^ The achieved correlation between the W fraction in the alloy and the PL emission energy is reported in Figure 8b.

Another common isoelectronic substituent for W in WS_2_ is chromium. Similarly to Mo_W_, Cr_W_ defects have a low formation energy^[^ ^38^, ^100^ ^]^ and can be unintentionally present in “as‐grown” MLs due to Cr impurities in the W precursor.^[^ ^38^, ^68^ ^]^ However, the electronic properties of Cr_W_ are markedly different from the Mo_W_ one.^[^ ^38^ ^]^ Ab initio simulations and experimental STS spectra reveal the presence of three unoccupied in‐gap defect states below the CBM. As visible in the STS spectra reported in Figure 8c, the lowest defect state is 400 meV below the CBM. On the contrary, the defect states at higher energies appear to be very similar, with a split of 80 meV, suggesting that their degeneracy is removed by spin‐orbit‐coupling.^[^ ^38^ ^]^ The Cr atom is strongly hybridized with the host material since the defect states are characterized by contributions of Cr 3d as well as W 5d and S 3p orbitals.^[^ ^38^ ^]^ The origin of these defect levels is not completely clear since Cr and Mo are isoelectronic, but the latter does not cause any defect states. It is supposed that strain associated with the smaller atomic radius of Cr with respect to Mo and W or the different energetics of 3‐d for Cr and 4‐d orbitals for Mo and W are responsible for the defect states.^[^ ^38^ ^]^ The optical properties of Cr_W_ have not been deeply analyzed yet, but it is believed that these defects act as effective radiative recombination centers and might host defect‐bound excitons.^[^ ^38^ ^]^ For example, single‐photon emission from Cr_W_ states has been recently achieved.^[^ ^48^ ^]^

#### 5.1.2 Non‐Isoelectronic n‐Doping Substituents

**Figure 9:**
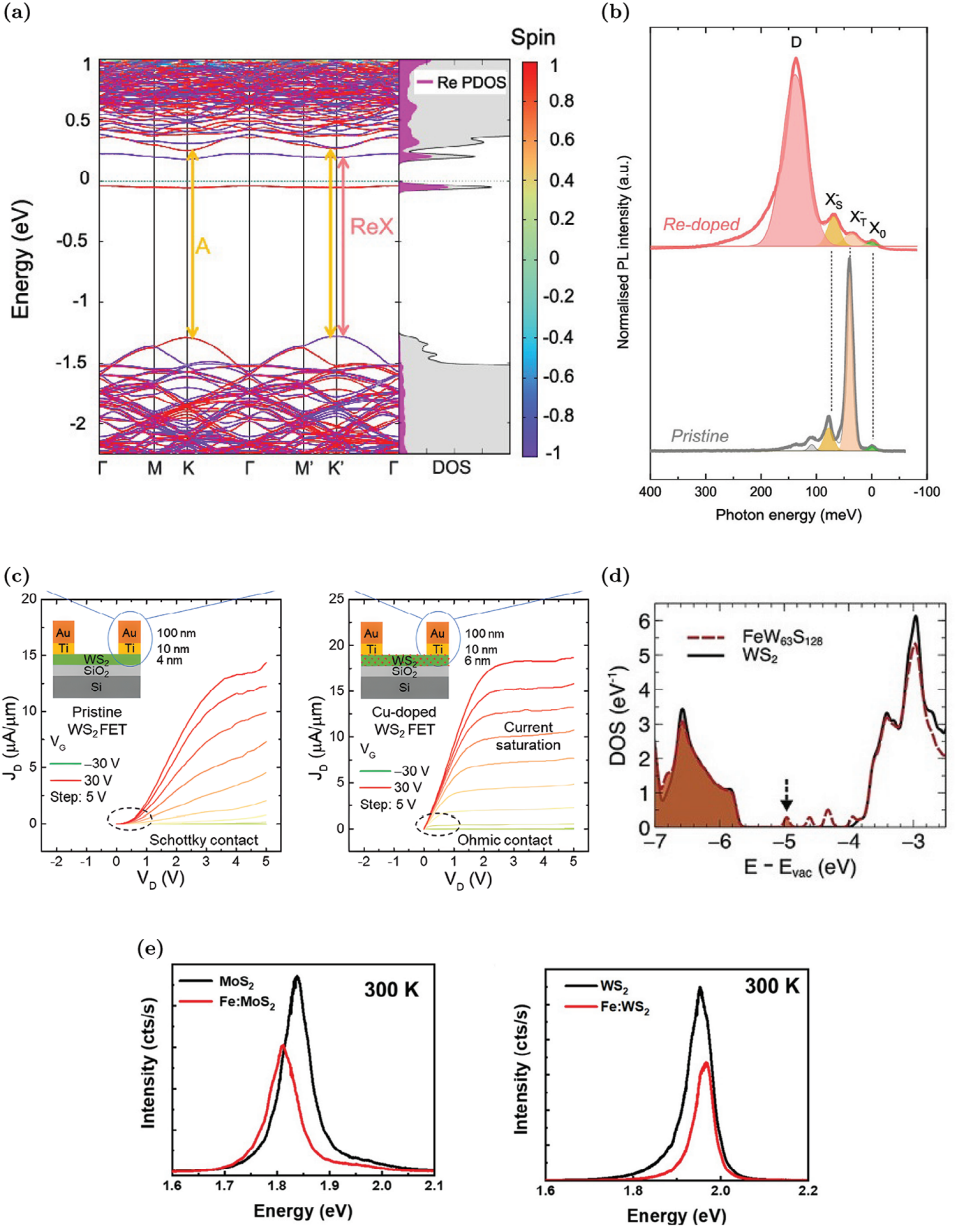
Effects of n‐type W substituents in WS_2_ MLs: a) Band structure and DOS for a (7 × 7) supercell with one Re_W_. The color scale in the band diagram indicates spin contributions for the states. Re PDOS and total DOS are reported in pink and gray, respectively. The Fermi level is set to zero. Reproduced with permission.^[^ ^95^ ^]^ Copyright 2021, American Chemical Society. b) PL spectra of Re‐doped and pristine ML at 4 K. The energy scale refers to the neutral exciton peak (X_0_) energy and the bound‐exciton peak is denoted with D. Reproduced with permission.^[^ ^95^ ^]^ Copyright 2021, American Chemical Society. c) Comparison of the output characteristics of pristine and Cu‐doped WS_2_ FETs at various gate voltages. Reproduced with permission.^[^ ^18^ ^]^ Copyright 2020, Royal Society of Chemistry. d) Comparison of DOS of Fe‐doped and pristine WS_2_. The energy reference is set to the vacuum level and the position of the Fermi level is identified by the black arrow. Reproduced with permission.^[^ ^100^ ^]^ Copyright 2021, Wiley‐VCH. e) Comparison of PL emission in Fe‐doped MoS_2_ and Fe‐doped WS_2_: Fe defect causes an exciton‐to‐trion conversion (red‐shift) in MoS_2_ and a trion‐to‐exciton conversion (blue‐shift) in WS_2_. Reproduced with permission.^[^ ^107^ ^]^ Copyright 2020, IOP publishing.

Different non‐isoelectronic transition metal atoms have been analyzed as W substituents to tune the carrier concentration in WS_2_. For example, rhenium atoms are expected to behave as n‐dopant in different TMD MLs due to their extra electron.^[^ ^104^, ^105^, ^106^ ^]^ Ab initio simulations confirm the presence of a first occupied level (spin‐up contribution) and a second empty state (spin‐down contribution) inside the gap with an evident Re contribution, as reported in the band diagram and in the DOS of **Figure** **9**a. These states are about 300 and 100 meV below the CBM, respectively.^[^ ^95^ ^]^ However, the occupied state is quite deep, and its ionization is unlikely at room temperature. Hence, differently from MoS_2_, Re_W_ is not an effective n‐dopant in WS_2_, as confirmed by electric‐transport measurements. ^[^ ^95^ ^]^ Nevertheless, also Re_W_ is appealing as SPE, since the unoccupied defect state can host a defect‐bound exciton. The associated energy transition is labeled as ReX in Figure 9a and is compared to the traditional band‐to‐band transition (A). As shown in the low temperature PL spectrum in Figure 9b, there is an evident peak at about 140 meV below the neutral exciton. This is associated to a Re_W_‐bound exciton and its high intensity is appreciable for the controlled emission of single photons.^[^ ^95^ ^]^

Due to the ineffectiveness of Re_W_ as n‐dopant in WS_2_, other transition metal atoms were investigated. For example, copper has been proposed as donor atom due to its five extra electrons with respect to W.^[^ ^108^ ^]^ Theoretical simulations confirm a favorable formation of Cu_W_ defects and the presence of different defect states close to the CBM that are partially occupied.^[^ ^108^ ^]^ In this case, the effectiveness of these donor levels is confirmed experimentally by electric‐transport measurements.^[^ ^18^ ^]^ Specifically, Cu doping is employed to reduce the WS_2_ work‐function and decrease the Schottky barrier at the metal‐WS_2_ contact from 120 to 90 meV.^[^ ^18^ ^]^ The quality of the ohmic‐contact is confirmed by the output characteristic of Cu‐doped WS_2_‐based FETs that has a perfectly linear characteristic at small drain voltages only in the case of Cu‐doping (Figure 9c).^[^ ^18^ ^]^

Another W substituent that is expected to behave as a donor dopant in TMD MLs as MoS_2_ is iron.^[^ ^20^, ^109^ ^]^ Nevertheless, a comparative study of Fe‐doped MoS_2_ and Fe‐doped WS_2_ reveals that Fe_W_ has opposite effects in these materials.^[^ ^107^ ^]^ The DOS analysis of Fe_W_ defect, reported in Figure 9d, shows that there is an occupied mid‐gap state and two unoccupied defect levels in the WS_2_ gap.^[^ ^100^ ^]^ However, the occupied donor level is supposed to be deeper in WS_2_ with respect to the correspondent one in MoS_2_, resulting in a low ionization probability for Fe_W_ donor defects.^[^ ^107^ ^]^ At the same time, the deep unoccupied Fe_W_ states are able to trap electrons, reducing the conduction band population.^[^ ^68^, ^107^ ^]^ Hence, Fe_W_ in WS_2_ is not a donor defect, but it actually decreases the electron concentration. This effect is also visible in the PL spectrum in Figure 9e: the decrease in the electron concentration causes a trion‐to‐exciton conversion, and totally the PL peak blue‐shifts. In addition, the PL intensity is also markedly suppressed due to the non‐radiative recombination processes mediated by the deep trap levels.^[^ ^68^, ^107^, ^110^ ^]^ Hence, Fe_W_ not only has an opposite role in WS_2_ with respect to MoS_2_, but it also has negative effects on the optoelectronic properties. These effects are even more detrimental considering that Fe_W_ defects tend to cluster in complexes in which there are also sulfur vacancies.^[^ ^68^, ^110^ ^]^

#### 5.1.3 Non‐Isoelectronic p‐Doping Substituents

**Figure 10:**
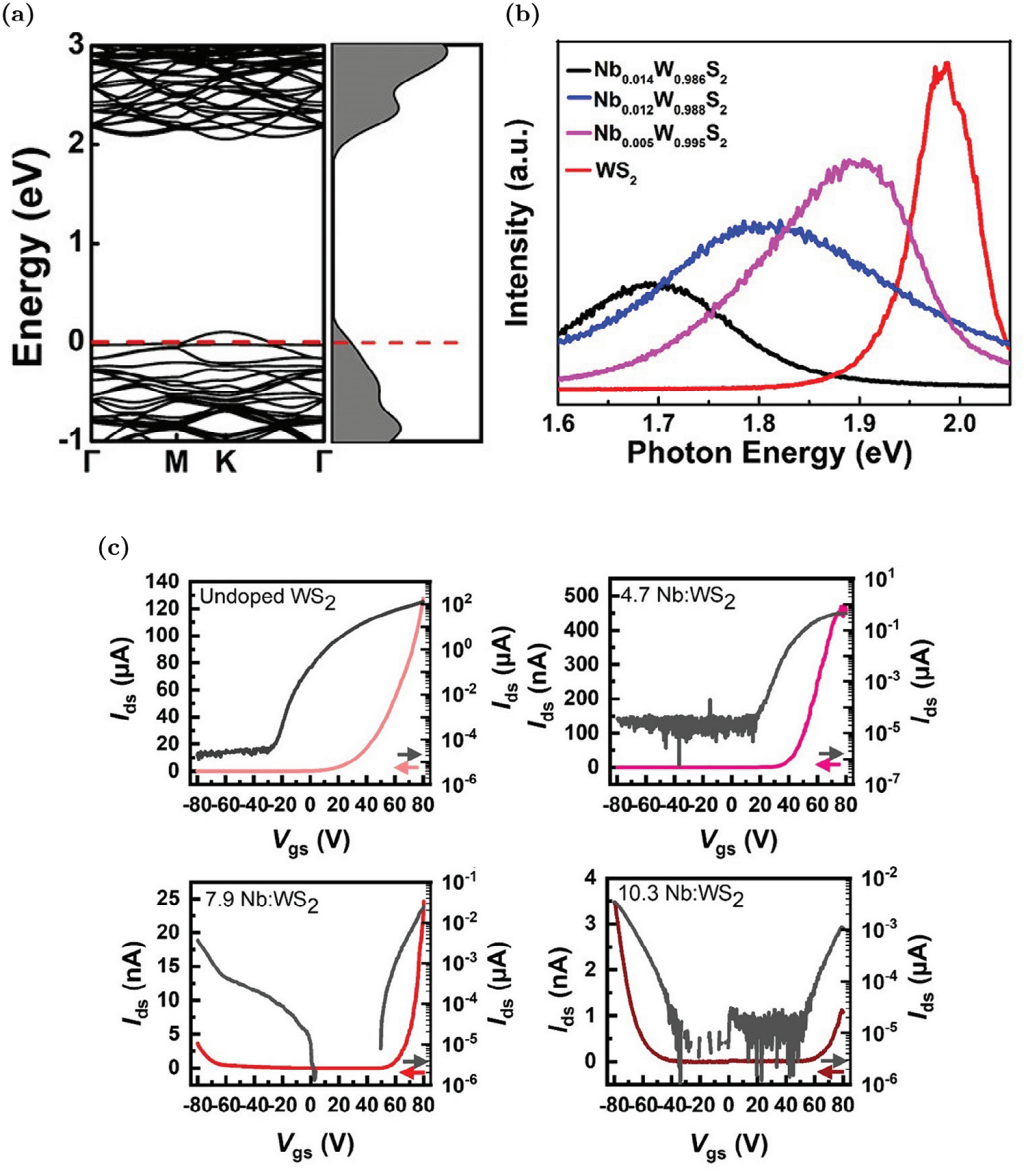
Properties of WS_2_ MLs with Nb_W_ substituent: a) Band diagram and DOS of Nb_W_. The Fermi level is identified by the red dashed line. Reproduced with permission.^[^ ^92^ ^]^ Copyright 2019, American Chemical Society. b) Variation of the PL spectra at increasing Nb_W_ concentration. Reproduced with permission.^[^ ^92^ ^]^ Copyright 2019, American Chemical Society. c) Transcharacteristics of FETs with progressively increasing Nb_W_ concentrations: Nb_W_ at 4.7% shows a decrease of the current associated to the n‐type transport (i.e., current branch at positive voltages), Nb_W_ at 7.9% results in an ambipolar device (i.e., co‐presence of current branches at positive and negative voltages) and Nb_W_ at 10.3% causes the predominance of the p‐type transport (i.e., current branch at negative voltages). Adapted with permission.^[^ ^111^ ^]^ Copyright 2019, American Chemical Society.

Considering that WS_2_ is intrinsically n‐type, W substituents that are able to decrease the intrinsic electron concentration and/or invert the majority carrier population are more appealing than the donor defects previously discussed. The most effective p‐type W substituent in WS_2_ and in other TMDs, is niobium.^[^ ^20^ ^]^ Different CVD recipes have been developed to achieve a tunable Nb concentration, adopting either Nb solid precursors, that are then evaporated,^[^ ^91^, ^92^ ^]^ or liquid precursors.^[^ ^111^ ^]^ Nb is easily incorporated in WS_2_ MLs in the W site, without lattice distortion, since WS_2_ and NbS_2_ are both stable in the 1‐H phase and have a similar lattice constant.^[^ ^92^ ^]^ Theoretical evaluations of the formation energy for Nb_W_ show that this defect is thermodynamically favorable.^[^ ^57^, ^92^, ^100^ ^]^ However, the synthesis parameters must be finely controlled to have a spatially uniform Nb_W_ distribution, since isolated Nb atoms tend to migrate and form clusters or line defects.^[^ ^91^, ^92^, ^111^ ^]^ Nb has been selected as substituent since it has one valence electron less than W, resulting in a p‐type dopant candidate. Ab initio simulation results, reported in **Figure** **10**a, show the formation of a resonant defect state due to Nb_W_, overlapped to the WS_2_ VBM. This pushes the Fermi level inside the valence band.^[^ ^91^, ^112^ ^]^ When the Nb_W_ concentration increases, additional defect states appear above the VBM, reducing the electronic gap.^[^ ^92^ ^]^ The effectiveness of this p‐dopant is confirmed by theoretical evaluations proving that Nb_W_ mainly exists as a negatively charged defect.^[^ ^57^ ^]^ Experimental evidence also confirms that the hole concentration increases due to the ionization of Nb_W_.^[^ ^91^, ^92^, ^111^ ^]^ For example, electric‐transport characterizations of FET devices show a progressively up‐shift of the threshold voltage, a decrease of the n‐type conduction branch at positive gate voltages and an enhancement of the p‐type branch at negative voltages when the Nb_W_ concentration rises.^[^ ^111^ ^]^ The conversion from n‐ to p‐type transport is reported in Figure 10c. Moreover, the contact resistance is also improved due to doping.^[^ ^92^ ^]^ Nb_W_ effects are also visible in the PL spectra: there is an enhancement of the exciton peak with respect to the trion one due to the electron concentration decrease.^[^ ^91^ ^]^ However, despite the trion‐to‐exciton conversion, the main PL variation reported in Figure 10b is an increasing red‐shift with the Nb_W_ concentration. This shift is caused by the decrease of the electronic gap associated to the formation of defect states close to the VBM.^[^ ^91^, ^92^, ^112^ ^]^

In addition to the well‐established p‐doping by Nb_W_, other W substituents have been proposed. For example, vanadium, similarly to Nb, has one valance electron less in comparison to W. The modulation of the electrical (conversion from n‐type to p‐type conduction) and optical properties (red‐shift of the PL peak due to acceptor levels in proximity of the VBM) are very similar to the Nb_W_ case.^[^ ^113^ ^]^ Nevertheless, vanadium defects tend to cluster and V_S_ defects are often present inside these clusters. Hence, the PL is progressively quenched as the vanadium density increases.^[^ ^113^ ^]^ P‐doping by indium substituent has also been reported.^[^ ^114^ ^]^ An In_W_ concentration equal to 6% is able to completely invert the majority carrier population and produce MLs in which the conduction is dominated by holes. In addition, at low In_W_ concentrations, it is possible to achieve a significant PL enhancement due to the trion‐to‐exciton conversion, as discussed in Section 2. Higher In_W_ concentrations can also introduce a positive trion peak, due to the complete n‐ to p‐type semiconductor conversion.

### 5.2 Sulfur Substituent Impurities

The substitution of sulfur atoms is a doping approach alternative to W replacement that offers several advantages. For example, chalcogen substitution causes minor lattice distortions compared to W substitution.^[^ ^20^ ^]^ Moreover, W substitution is achieved during the synthesis, while the S substitution can be performed also after the synthesis, resulting in an additional degree of freedom since the sample growth and the doping strategies are decoupled.^[^ ^20^ ^]^ Post‐growth doping by S substitution can be applied to WS_2_ MLs regardless of the synthesis methods and allows for a spatially selective doping.^[^ ^51^ ^]^ Similarly to the W substituent, the S one can be isoelectronic, n‐type or p‐type dopant.^[^ ^20^ ^]^

#### 5.2.1 Isoelectronic S Substituents

**Figure 11:**
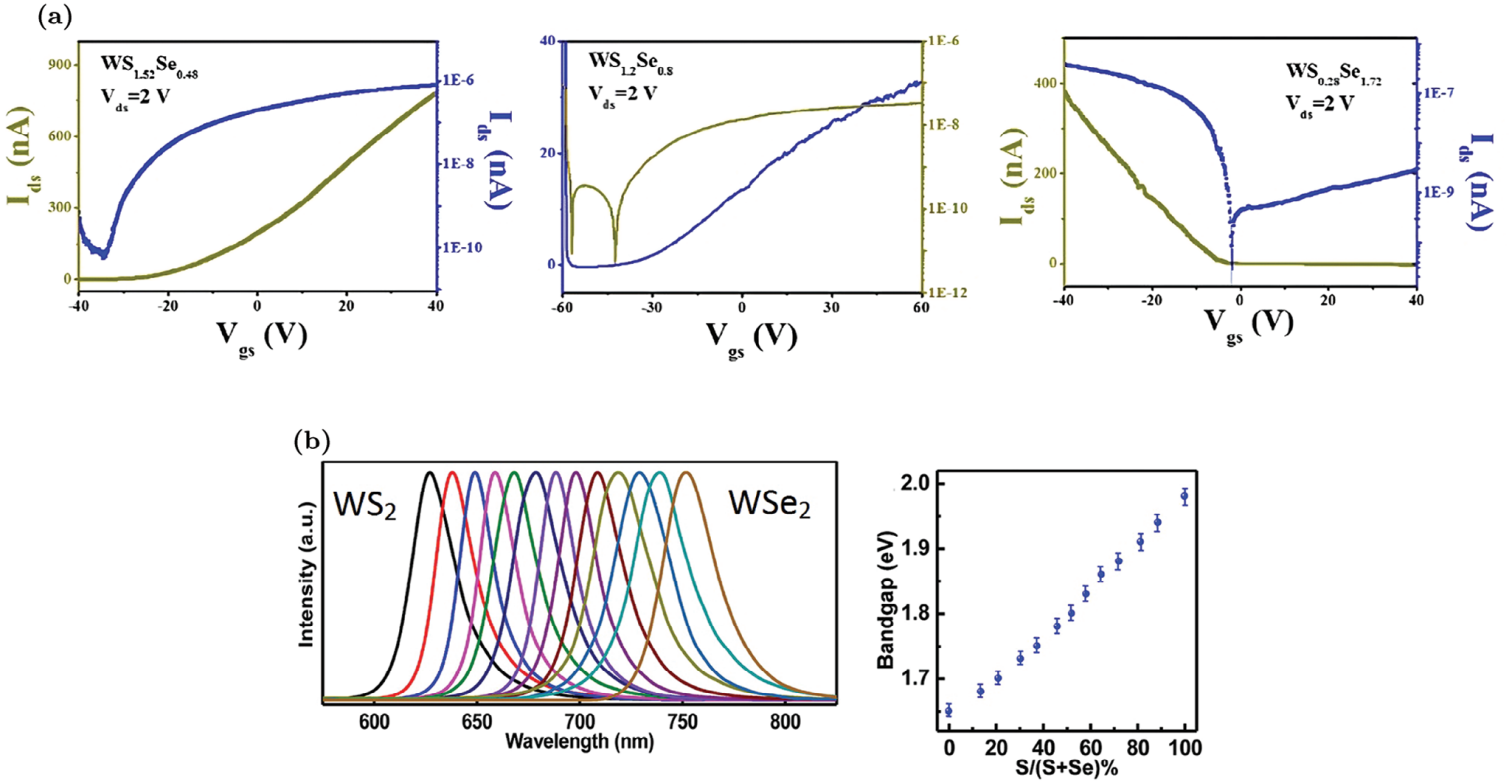
Properties of WS_2*x*_Se_2−2*x*_ alloys: a) Transcharacteristics of FETs at different S fraction (2*x* = 1.52, 1.2, and 0.28) with a systematic shift in carrier type, from n‐type behavior in the S‐rich phase to p‐type semiconductors in the Se‐rich phase. Reproduced with permission.^[^ ^116^ ^]^ Copyright 2021, Elsevier. b) Optical properties of MLs with different S fraction: normalized PL spectra of a series of alloys and correlation between optical gap and S ratio. Reproduced with permission.^[^ ^115^ ^]^ Copyright 2016, American Chemical Society.

The most common isoelectronic substituent for S is oxygen, as previously discussed in Section 4.2. Other types of isoelectronic atoms that are intentionally incorporated to achieve WS_2_‐based alloys are selenium and tellurium. For example, the synthesis of different ML samples of WS_2*x*_Se_2‐2*x*_ is commonly reported in the literature.^[^ ^115^, ^116^ ^]^ By means of CVD, it is possible to realize alloys with a tunable concentration of Se and a uniform distribution of substituent atoms.^[^ ^116^ ^]^ Since WS_2_ is intrinsically n‐type, while WSe_2_ is p‐type, alloys are a powerful strategy to tune the carrier polarity of the samples. By varying the S composition, it is possible to have p‐type (S fraction 0 ÷ 0.4), ambipolar (S fraction 0.4 ÷ 0.65) or n‐type (S fraction 0.65 ÷ 1) FETs, as shown in the transcharacteristics reported in **Figure** **11**a.^[^ ^115^, ^116^ ^]^ Moreover, due to the different gap, it is possible to linearly tune the PL emission peak position of the alloys, from about 630 nm (pure WS_2_) to 760 nm (pure WSe_2_), by changing the chalcogen fraction, as shown in Figure 11b.^[^ ^115^, ^116^ ^]^ Notice that the peaks remain sharp for all the compositions, proving the crystalline quality of the alloys.

A similar approach has also been adopted to synthesize WS_2*x*_Te_2−2*x*_ alloys.^[^ ^117^ ^]^ However, in this case, the tuning of the alloy properties is more challenging, considering that WS_2_ is stable in the semiconductor 1‐H phase, while WTe_2_ is stable in the semi‐metallic 1‐T′ one. Hence, the synthesis conditions are critical in order to control the phase transition. When the Te fraction is in the range 0 ÷ 0.5, the alloy is a semiconductor with an increasing p‐type behavior as the Te concentration rises. The alloy is semi‐metallic with quenched PL when the Te ratio exceeds 50%.^[^ ^117^ ^]^

#### 5.2.2 Non‐Isoelectronic n‐Doping S Substituents

**Figure 12:**
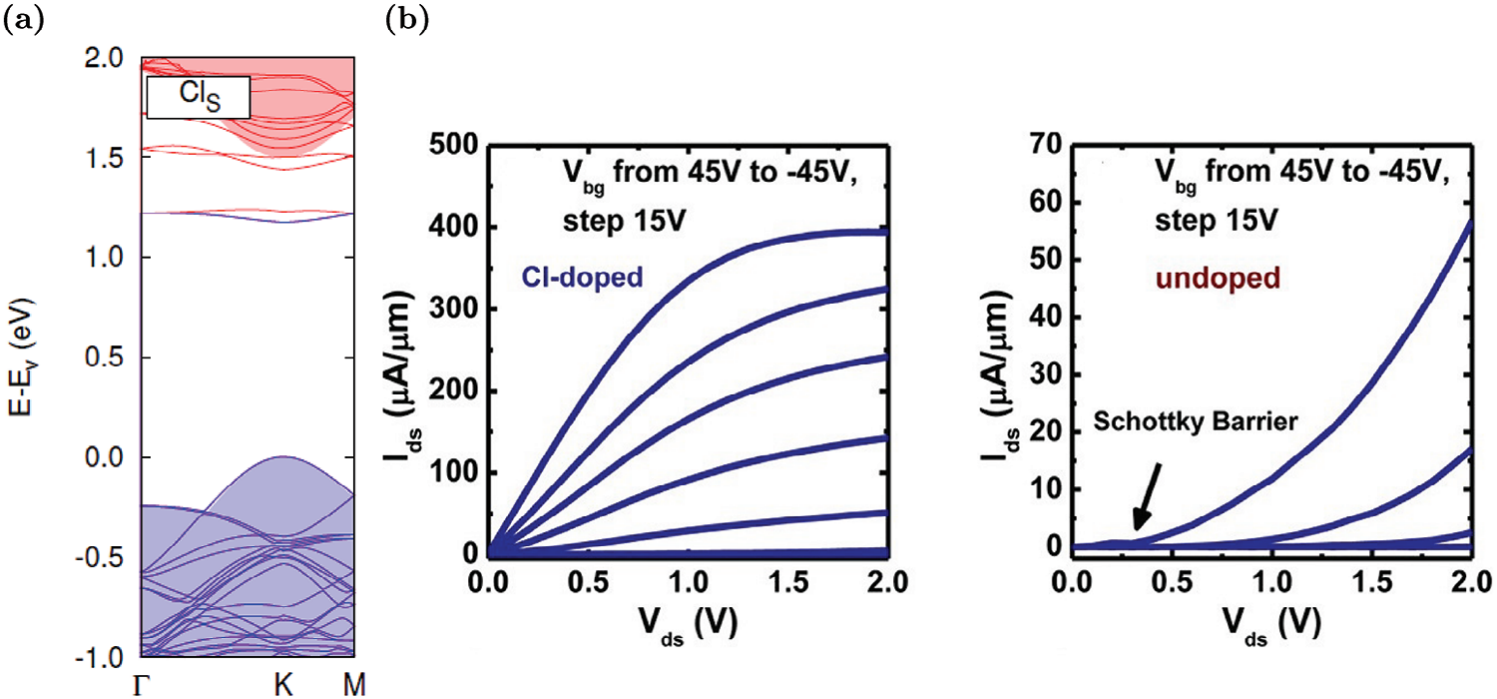
Properties of WS_2_ MLs with Cl_S_ substituents: a) Band diagram of Cl_S_, the energy reference is set to the valence band maximum, occupied and unoccupied states are depicted in blue and red, respectively, the band structure of the pristine ML is represented by the shaded areas. Reproduced with permission.^[^ ^119^ ^]^ Copyright 2014, American Physical Society. b) Comparison of the output characteristics of Cl_S_‐doped and pristine WS_2_ FETs. Reproduced with permission.^[^ ^118^ ^]^ Copyright 2014, American Chemical Society.

Considering non‐isoelectronic defects, the most interesting S substituent atoms that induce n‐doping in WS_2_ MLs are halogens, such as F, Cl, Br, and I.^[^ ^57^ ^]^ Indeed, they have an extra valence electron with respect to S. Among the halogen species, chloride doping is the most promising: Cl and S atoms have a similar atomic radius.^[^ ^57^, ^118^ ^]^ Different strategies for Cl incorporation are proposed, relying on sodium halide‐assisted CVD^[^ ^88^ ^]^ or post‐growth soakings in chloride based solutions.^[^ ^118^ ^]^ The stability of Cl doping in time is confirmed by theoretical^[^ ^57^ ^]^ as well as experimental^[^ ^118^ ^]^ evidence. The origin of the n‐type effect of Cl_S_ has been investigated by means of ab initio simulations that prove the existence of two couples of defect states below the CBM, as reported in the band diagram of **Figure** **12**a. These states originate from the hybridization of Cl 3p and W 5d orbitals.^[^ ^119^, ^120^ ^]^ The lowest‐energy couple is partially occupied and acts as donor level, while the other one is very shallow and completely unoccupied. Simulation results show that Cl_S_ is favorable as a positive charged defect (i.e., ionized donor).^[^ ^57^ ^]^ Other effects associated to Cl_S_ are the decrease of the gap due to the unoccupied shallow defect states^[^ ^120^ ^]^ and the capability to passivate the S vacancies.^[^ ^88^, ^118^, ^121^ ^]^ The beneficial effects of Cl_S_ are mainly exploited to improve the electric‐transport properties. Both theoretical analysis^[^ ^120^ ^]^ and experimental evidences^[^ ^118^ ^]^ show that the n‐doping due to Cl_S_ is able to effectively decrease the Schottky barrier (drop of about 0.1 eV for a Ni/Au electrode) at the metal‐WS_2_ contact. The resulting 2 ÷ 3 order of magnitude suppression of the contact resistance is attributed to the decrease of the WS_2_ work‐function due to doping, as well as to the passivation of the S vacancies that can pin the Fermi level at the interface with the metal.^[^ ^118^ ^]^ The improvement of the contact region is clearly visible in the output characteristic reported in Figure 12b, where a linearity at small drain voltages and a sixfold increase of the on‐state current in case of Cl doping is reported.^[^ ^118^ ^]^ The Cl_S_ doping has also beneficial effects on the optical properties. Similarly to the MoS_2_ case, it is expected that Cl_S_‐doped WS_2_ MLs are characterized by a PL enhancement due to the V_S_ passivation and a red‐shift of the emission due to the decrease of the electronic gap caused by the shallow states. A red‐shift is also expected due to the exciton‐to‐trion conversion associated to the electron concentration increase, as discussed in Section 2.^[^ ^88^ ^]^

#### 5.2.3 Non‐Isoelectronic p‐Doping S Substituents

**Figure 13:**
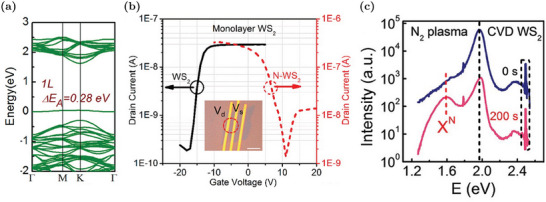
Properties of WS_2_ MLs with N_S_ substituents: a) Band structure of N_S_ doped WS_2_, energies are referred to the Fermi level and Δ*E*_A_ is the acceptor ionization energy. Reproduced with permission.^[^ ^93^ ^]^ Copyright 2017, American Chemical Society. b) Comparison of the transcharacteristics of pristine and N_S_‐doped WS_2_ FETs. The inset is the optical image of the FET, scale bar is 5 μm. Reproduced with permission.^[^ ^51^ ^]^ Copyright 2018, American Chemical Society. c) Comparison of the PL spectra of ML exposed to remote N_2_ plasma for different time: pristine (*t* = 0 s) and N_S_‐doped (*t* = 200 s) samples. Reproduced with permission.^[^ ^52^ ^]^ Copyright 2022, American Chemical Society.

If halogen species are used as n‐dopant substituents in WS_2_, group‐V elements, such as nitrogen and phosphorus, are employed as p‐dopants since they have one valence electron less with respect to S.^[^ ^122^ ^]^ Among these chemical species, the N_S_ defect is the most analyzed.^[^ ^50^, ^51^, ^52^, ^67^, ^93^ ^]^ N_S_ is obtained either directly during CVD^[^ ^93^ ^]^ or, more commonly, after the synthesis by means of short N_2_ plasma treatments^[^ ^50^, ^67^ ^]^ or exposure to nitrogen radicals.^[^ ^51^ ^]^ The N incorporation is successful due to the low formation energy of N_S_^[^ ^122^ ^]^ and its effects on the WS_2_ properties are stable over time and at high temperatures.^[^ ^51^, ^93^ ^]^ Moreover, the beneficial effects are also reproducible for large scale applications.^[^ ^93^ ^]^ Ab initio analyses relate the origin of the p‐doping associated to N_S_ to a partially occupied couple of defect states close to the VBM, as shown in **Figure** **13**a.^[^ ^93^ ^]^ At increasing N_S_ concentration, additional defect states appear above the VBM.^[^ ^51^ ^]^ The N_S_ defect states derive from the hybridization of N 2p, W 5d, and S 3p orbitals.^[^ ^51^, ^122^ ^]^ The unoccupied state is sufficiently shallow (about 0.24–0.28 eV above the VBM) to act as an effective acceptor level.^[^ ^51^, ^93^ ^]^ This thesis is also confirmed by electric‐transport measurements on N‐doped WS_2_‐based FETs.^[^ ^51^, ^93^ ^]^ By tuning the N_S_ concentration, it is possible to decrease the intrinsic electron concentration^[^ ^67^ ^]^ and achieve population inversion with a predominance of holes.^[^ ^51^, ^93^ ^]^ Hence, the transcharacteristic of N_S_‐doped WS_2_‐based FETs, visible in Figure 13b, are similar to the current‐voltage curves of samples with other p‐dopant defects such as Nb_W_ and Se_S_.

The effects of N_S_ are also visible in the PL emission spectrum. The main peak slightly red‐shifts due to the shallow defect states that reduce the electronic gap.^[^ ^50^ ^]^ Moreover, the PL intensity tends to increase due to the passivation of V_S_ by N atoms and due to the trion‐to‐exciton conversion. However, the peculiarity of N_S_ with respect to other S substituents is its applicability in single‐photon emission. The PL spectrum is characterized by a low energy peak (X^N^) associated to N_S_ defects.^[^ ^52^ ^]^ As visible in Figure 13c, this peak falls at 1.59 eV, being well separated from the main emission peak at about 2 eV. Moreover, it is also well distinguishable from the defect‐bound excitons associated to V_S_ at about 1.8 eV. From a detailed analysis of all possible radiative paths, it is supposed that the X^N^ peak is associated to the radiative transition from the conduction band minimum to the defect state of N_S_ when negatively charged.

In addition to S substitutions by single atoms, it is possible to achieve p‐doping by means of carbon functional group substitutions.^[^ ^123^ ^]^ For example, the CH group acts as an acceptor substituent replacing an S atom. The CH group can be unintentionally present in “as‐grown” samples, with variable density (10^10^ ÷ 10^12^ cm^‐2^), due to the presence of hydrocarbon impurities in the synthesis environment.^[^ ^63^ ^]^ CH can be also intentionally incorporated via exposure to methane‐based plasma.^[^ ^123^ ^]^ Recorded STS spectra and ab initio simulations identify a couple of defect states overlapped with the valence band maximum that can be populated by valence electrons, resulting in effective acceptor levels.^[^ ^38^, ^123^ ^]^ This type of defect is commonly identified as a negative charged site (i.e., ionized acceptor).^[^ ^38^ ^]^ CH‐doping results in an effective method to control the carrier concentration in WS_2_ MLs, because it is possible to decrease the intrinsic electron concentration to get ambipolar o p‐type transistors, by simply modulating the CH_4_ plasma flow.^[^ ^123^ ^]^ Nonetheless, this type of defect has negative consequences on the emission properties. It causes a red‐shift of the PL peak (due to the gap decrease associated to the shallow states) and, above all, an evident PL suppression and broadening due to the extended removal of S atoms.^[^ ^123^ ^]^

### 5.3 SCTD by Adatoms

Doping by adatom adsorption is a general approach that can be applied to a wide range of atomic species, considering both metal^[^ ^124^ ^]^ and non‐metal^[^ ^125^ ^]^ elements. Considering that each species is characterized by its own atomic radius and chemical affinity, different adatoms can be adsorbed on different sites on the WS_2_ ML.^[^ ^99^ ^]^

**Figure 14:**
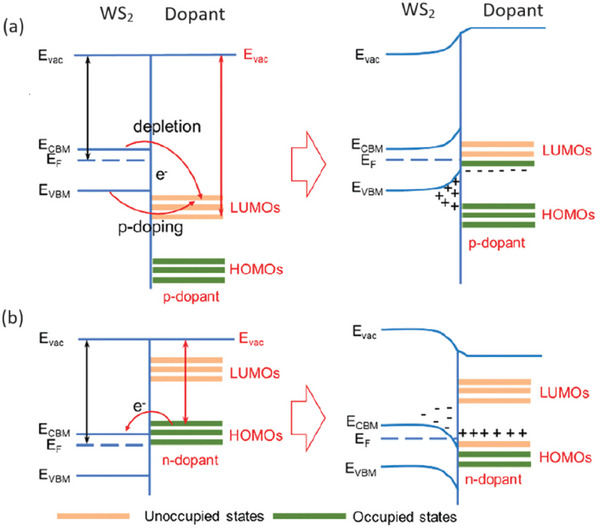
Schematic energy levels for the SCTD mechanism in case of a) p‐ and b) n‐type adsorbed dopant, before (left) and after (right) the charge transfer. E_VAC_, E_CBM_, E_F_, and E_VBM_ correspond to the vacuum level, the conduction band minimum, the Fermi level, and the valence band maximum of WS_2_ ML, respectively. The highest occupied states and the lowest unoccupied states of the adsorbed species (dopant) are indicated as HOMOs and LUMOs, following the molecular orbital nomenclature. Adapted with permission.^[^ ^90^ ^]^ Copyright 2018, Royal Society of Chemistry.

The process of SCTD by adatoms as well as adsorbed molecules is driven by the energy level alignment between the bands of the semiconductor and the highest occupied/lowest unoccupied states of the adsorbate, as schematically represented in **Figure** **14**.^[^ ^90^, ^126^ ^]^ Comparing the band diagram of pristine WS_2_ ML and the energy spectrum of the isolated adsorbate, if the lowest empty state of the adsorbate is slightly below the VBM, there is an electron transfer from the valence band to the state of the adsorbate. The result is an increase of the hole concentration in the WS_2_ valence band and the adsorbate can be classified as an acceptor species for WS_2_ ML. On the contrary, if the highest occupied state of the adsorbate is above the CBM, there is an electron transfer from the state of the adsorbed species to the conduction band, increasing the free electron concentration of the WS_2_ ML (donor species).^[^ ^90^ ^]^ For more details about the SCTD processes, the interested readers can refer to specific reviews, for example, refs. [90, 126].

As discussed in Section 4.4, H can be adsorbed unintentionally as adatom over the S atom. In addition, other species have also been reported as adatom impurities. For example, it is known that sodium contaminants are commonly present on the surface of SiO_2_/Si wafers.^[^ ^127^ ^]^ Moreover, Na compounds (e.g., sodium halides) are commonly adopted as promoters in CVD synthesis, since the Na atom favors the formation of larger flakes.^[^ ^88^, ^92^ ^]^ Hence, it is likely that some Na atoms are trapped between the ML and the substrate. Due to the low ionization energy, Na adatoms easily release electrons to the WS_2_ ML. For that reason, some works propose Na adatoms as a further source of the electrons that increases the intrinsic carrier concentration in WS_2_.^[^ ^87^ ^]^ Theoretical simulations confirm that Na adatoms are able to generate donor occupied defect states close to the CBM and induce a metallic behavior.^[^ ^128^ ^]^

In addition to H and Na adatoms, mainly adsorbed during the ML synthesis, it is possible to dope WS_2_ via post‐growth treatments. The most common intentional adatom doping employs halogens such as fluorine^[^ ^17^ ^]^ and chlorine.^[^ ^129^ ^]^ These species are adsorbed preferentially over the S atom. It should be noted that the effect of halogen dopants differs when they replace sulfur atoms compared to when they are adsorbed on the ML.^[^ ^17^ ^]^ As substituents, the n/p dopant nature is determined by the additional/missing valence electrons of the dopant in comparison to S atom. Hence halogens act as donor in this configuration. On the contrary, in case of SCTD, the high electronegativity of the halogens with respect to the S atom, allows adatoms to attract and localize electrons, reducing the free carrier concentration of WS_2_. This is confirmed by ab initio simulations that identify a donor level below the conduction band minimum in the case of Cl_S_ and an acceptor level above the valence band maximum in the case of Cl adatom.^[^ ^129^ ^]^

**Figure 15:**
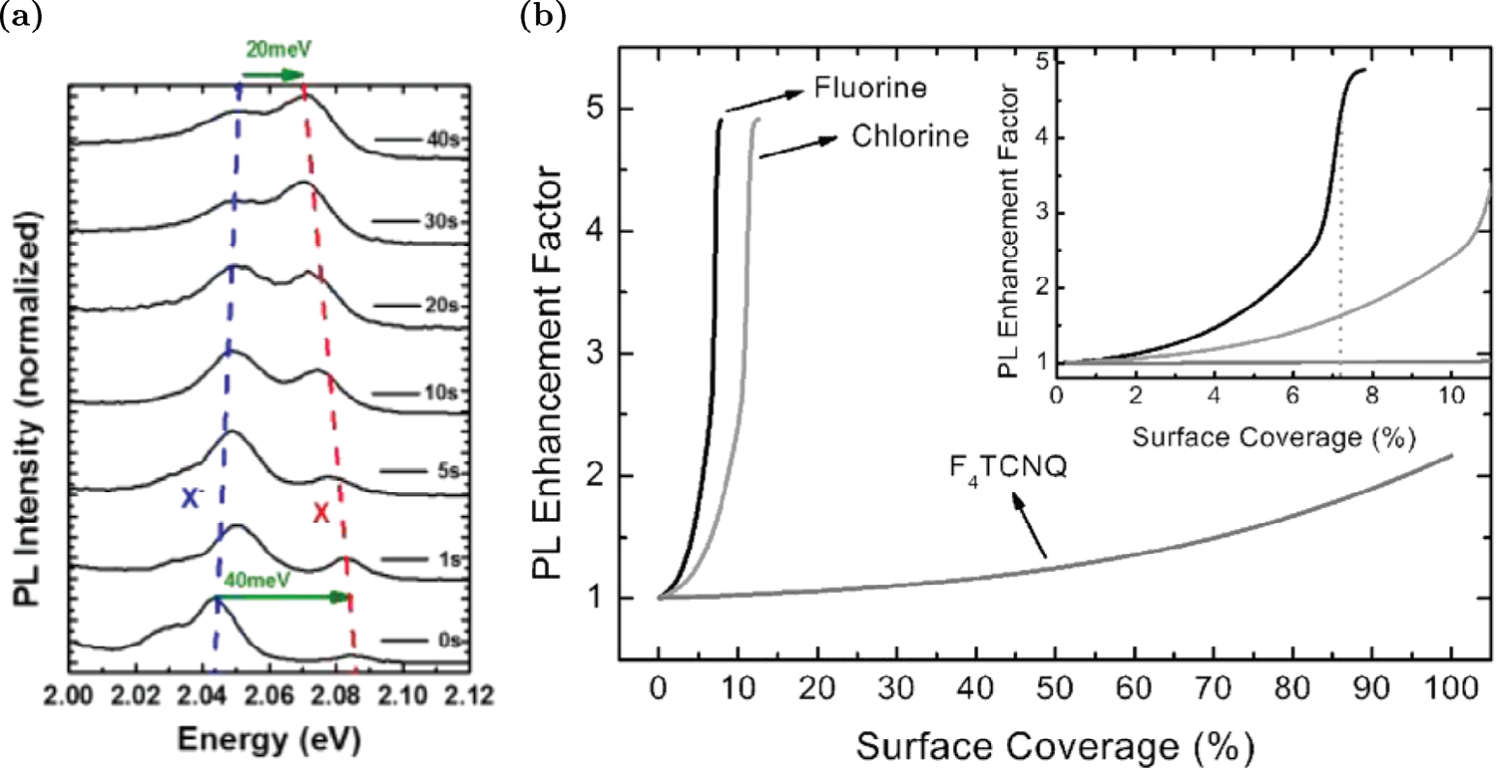
Effects of adatom adsorption on WS_2_ MLs: a) Evolution of the PL spectrum with the photoclorination time: X and X^−^ refer to the neutral and the trion peak, respectively. Reproduced with permission.^[^ ^129^ ^]^ Copyright 2018, IOP Publishing. b) Enhancement factors of PL efficiency as a function of the surface coverage of various dopants (F adatom, Cl adatom, and adsorbed F_4_TCNQ). The curves are shown only up to the complete trion suppression. Reproduced with permission.^[^ ^17^ ^]^ Copyright 2016, Wiley‐VCH.

Cl adatom doping is usually achieved through exposure to Cl_2_ gas under UV laser pulses, which dissociate the halogen molecules.^[^ ^129^ ^]^ This post‐growth laser exposure approach allows for a spatially selective modification of the material properties. At the same time, the effects of the photochlorination are completely reversible upon continuous‐wave (CW) laser rastering. It is then possible to induce the Cl atom desorption in a controllable way and recover the original properties. The most beneficial effect of photochlorination is an evident PL enhancement: this effect is achieved due to the complete trion‐to‐exciton conversion.^[^ ^129^ ^]^ The evolution of the PL spectrum (peak intensity and shift) as the photoclorination time increases, reported in **Figure** **15**a, is compatible with the trend reported in Figure 1b at decreasing electron concentration. Indeed, an electron density variation of about 3 10^12^ cm^‐2^ after only 40 s of photochlorination is achieved.^[^ ^129^ ^]^ The Cl adatom doping is also proposed as a strategy to tune the valley polarization and control the degree of polarization of the PL emission.^[^ ^121^ ^]^

Similar doping effects are achieved via fluorination through fluorine plasma.^[^ ^17^ ^]^ However, the high adsorption energy of the F adatoms with respect to the Cl ones results in a larger electron transfer. Hence, the PL enhancement due to F adatoms is more effective with respect to Cl and other acceptor molecule adsorption.^[^ ^17^ ^]^ For example, comparing F, Cl adatom and a common acceptor molecule (F_4_TCNQ), in Figure 15b, the maximum PL enhancement due to the complete trion peak suppression is achieved with only 7.2% of F adatom coverage, while Cl and F_4_TCNQ require higher concentrations to reach similar results. Moreover, differently from other SCTD approaches, the property modifications due to F adatoms are also quite stable in time.^[^ ^17^ ^]^ Nevertheless, complete reversibility of the fluorination effects is still possible via H plasma. A main drawback of the fluorination approach is the use of the fluorine base chemistry which can cause unwanted etching effects at high F concentrations.^[^ ^17^ ^]^

### 5.4 SCTD by Molecule Adsorption

Similarly to adatom doping, molecules adsorbed on the basal plane of WS_2_ MLs can alter the carrier concentration due to surface charge transfer.^[^ ^90^ ^]^ In certain studies, the samples are intentionally exposed to specific target gases to achieve the modulation of the electronic and optical properties of WS_2_ through molecule adsorption, thus enabling gas sensing.^[^ ^130^, ^131^ ^]^ In other cases, the interaction between the ML and molecules is unintentional and occurs as a result of the unavoidable exposure to common environmental gases like O_2_ and water vapors.^[^ ^132^ ^]^ Finally, the intentional adsorption of specific molecules, such as superacids^[^ ^133^ ^]^ and thiolated compounds,^[^ ^134^ ^]^ is exploited to designedly modulate the material properties through SCTD. In general, molecules are only physisorbed on the basal plane of the MLs, due to the high stability of the surface in 2D materials.^[^ ^98^, ^135^ ^]^ However, the presence of defects such as S vacancies allows chemisorption processes, increasing the charge transfer and/or passivating the vacancies.^[^ ^135^ ^]^ Hence, molecule adsorption is also employed to passivate the vacancy sites and improve the material properties.^[^ ^62^, ^66^, ^136^ ^]^

#### 5.4.1 Gas Molecule Adsorption

**Figure 16:**
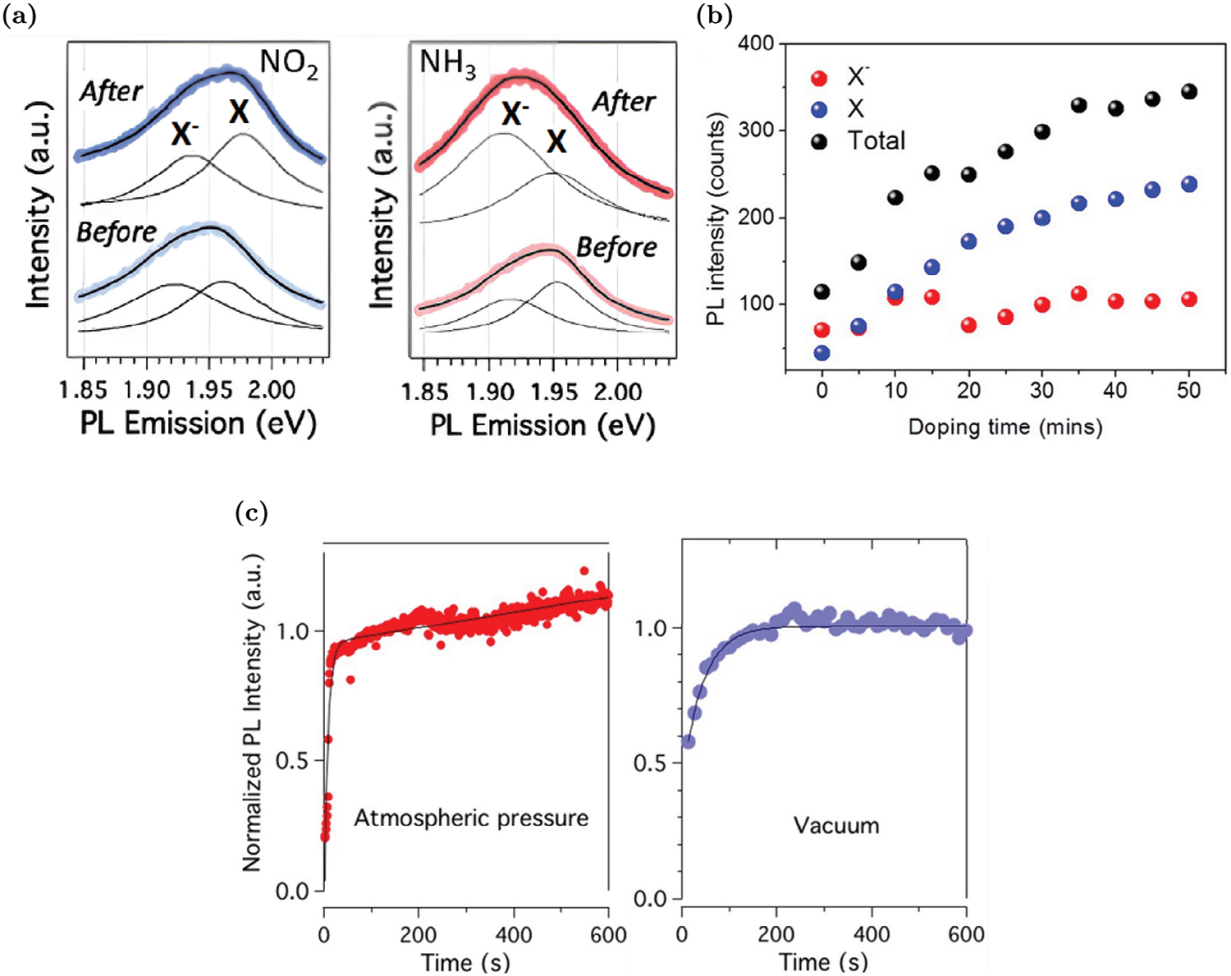
Effects of molecule adsorption on WS_2_ MLs: a) PL emission spectra collected before and after the exposure to different gases (NO_2_ and NH_3_). Neutral exciton and negative trion peaks are labeled “X” and “X^−^”, respectively. Adapted with permission.^[^ ^139^ ^]^ Copyright 2021, Royal Society of Chemistry. b) Variation of the integrated PL intensity of trion (X^**−**^) and exciton (X) components at increasing exposure time to water. Reproduced with permission.^[^ ^55^ ^]^ Copyright 2014, American Chemical Society. c) Evolution of PL intensity over laser irradiation time performed at atmospheric pressure and under vacuum. Adapted with permission.^[^ ^54^ ^]^ Copyright 2019, IOP Publishing.

A large number of molecules have been investigated to understand the effects of the adsorption on WS_2_ in view of gas‐sensing applications.^[^ ^98^, ^137^, ^138^ ^]^ Here, we summarize the effects of this large set of molecules, reporting comparative studies of a donor (NH_3_) and an acceptor molecule (NO_2_).^[^ ^139^ ^]^ Considering that WS_2_ MLs are intrinsically n‐type, acceptor molecules as NO_2_ can easily withdraw electrons. There is substantial evidence indicating that the resistivity of WS_2_ samples increases upon exposure to a small amount (also tens of parts per billion) of NO_2_ due to the decrease in the electron concentration.^[^ ^140^, ^141^ ^]^ On the contrary, pristine WS_2_ is less influenced by donor molecules as NH_3_. Indeed, it is unlikely for a system that is already inherently electron‐rich, to acquire additional electrons from the adsorbed species.^[^ ^17^ ^]^ For that reason, the sensitivity toward NH_3_ adsorption is more evident in p‐doped samples. For example, in samples with F adatoms (acceptor defects), the electron concentration almost doubles upon exposure to 10 ppm of NH_3_.^[^ ^17^ ^]^ The variation of carrier concentration due to donor/acceptor molecules has also evident impacts on the PL emission of WS_2_.^[^ ^139^ ^]^ Experimentally, flakes exposed to NO_2_ are characterized by a blue‐shift of the PL emission, as reported in **Figure** **16**a. As discussed in Section 2, this shift is due to the trion‐to‐exciton conversion caused by a reduction of the electron concentration. On the other hand, NH_3_ adsorption is responsible for an increase in the electron concentration and the PL peak red‐shifts due to the exciton‐to‐trion conversion (Figure 16a).

#### 5.4.2 Oxygen and Water Adsorption

Special attention is given to O_2_ and H_2_O, considering that WS_2_ flakes are frequently exposed to these gases during synthesis, handling, characterization and storage. Both molecules are expected to be acceptors for WS_2_.^[^ ^98^ ^]^ This is clearly proven by the PL analysis of WS_2_ samples covered by a droplet of water. As visible in Figure 16b, increasing the H_2_O adsorption time, the exciton peak is significantly enhanced with respect to the trion one and the total PL intensity increases, as expected for withdrawing molecules.^[^ ^55^ ^]^ Notice that this effect is completely reversible upon low temperature annealing, showing that water molecules are physisorbed. Similar effects are also observed for samples in O_2_ controlled environment.^[^ ^27^ ^]^ In addition, H_2_O and O_2_ molecules are also able to dynamically change the PL spectra during the laser exposure.^[^ ^132^ ^]^ The energy provided by the laser source can induce the desorption of organic contaminants from the ML surface, exposing new adsorption sites for the molecules present in the environment (i.e., mainly H_2_O and O_2_). Hence, the PL enhancement upon laser exposure in an atmospheric environment is related to the trion‐to‐exciton conversion due to physisorbed acceptor H_2_O and O_2_ molecules.^[^ ^142^ ^]^ However, strain ^[^ ^42^ ^]^ or defects as V_S_^[^ ^62^ ^]^ can also induce chemical reactions activated by the laser power between adsorbed molecules and the basal plane of WS_2_. Hence, the PL enhancement during the laser exposure in the ambient atmosphere can be attributed to the passivation of S vacancies by O_2_ chemisorption at the defect sites.^[^ ^142^ ^]^ As shown in the PL spectra in Figure 16c, a significant PL enhancement is achieved only when the MLs are irradiated in ambient atmosphere, as the presence of O_2_ in the case of in‐vacuum conditions is insufficient for effective V_S_ passivation. Considering the previously discussed beneficial effects of V_S_ passivation by oxygen incorporation, intentional O_2_ chemisorption becomes a promising approach to heal the WS_2_ and increase the optical emission.^[^ ^53^, ^62^ ^]^ However, differently from O_S_, chemisorbed O_2_ molecules in the V_S_ sites cause a local decrease of the electronic gap, resulting in a slight red‐shift of the PL spectrum.^[^ ^62^, ^132^ ^]^ In addition, the prolonged laser exposure results in an excessive replacement of S with O, and large areas of the material are oxidized, causing a severe PL quenching.^[^ ^143^ ^]^

#### 5.4.3 Organic Molecule Adsorption

**Figure 17:**
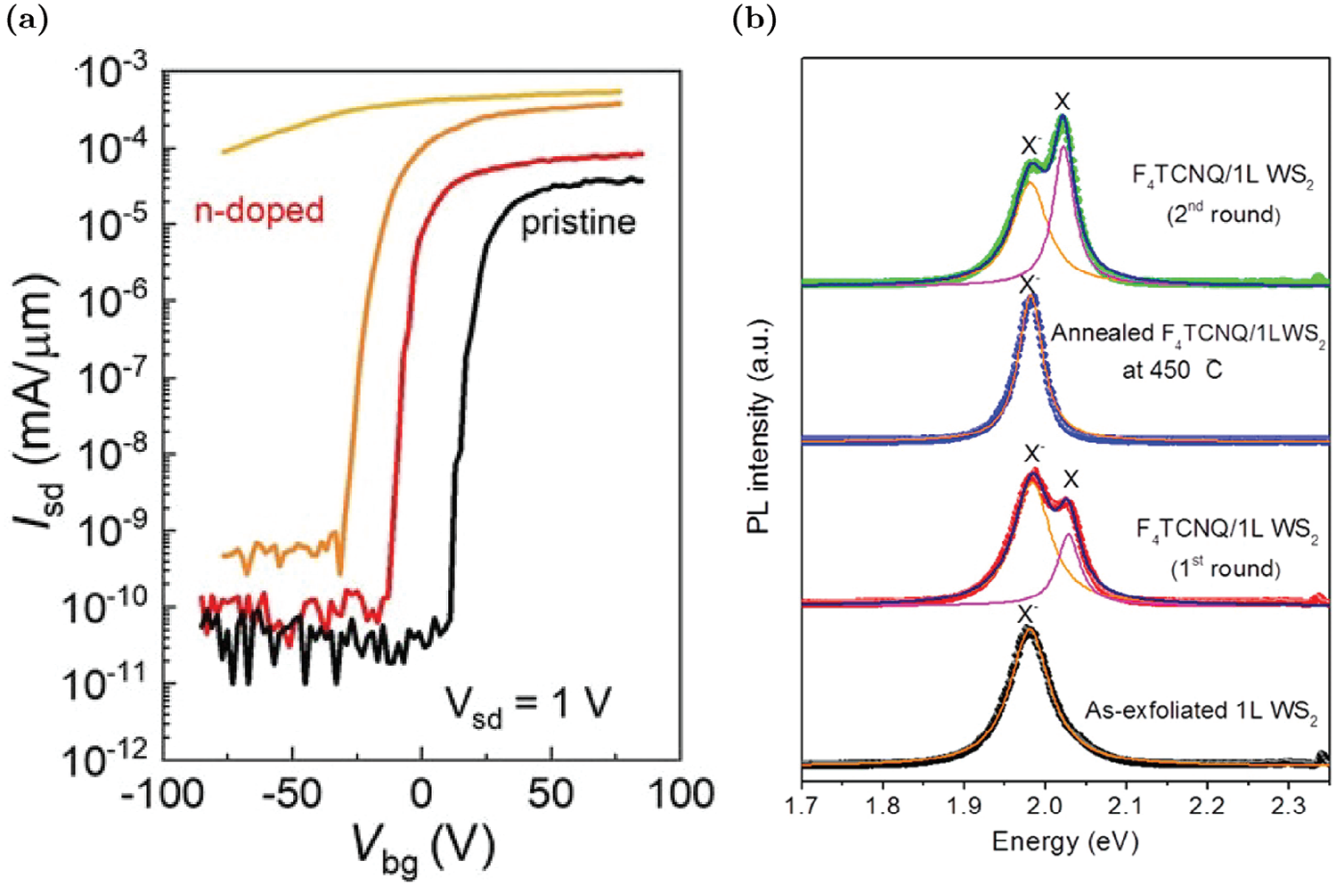
Effects of organic molecule adsorption on WS_2_ MLs: a) Transcharacteristics of FETs at room temperature before (black), after 30 s (red), 1 min (orange), and 10 min (yellow) of n‐dopant (RhCpCp^*^)_2_ treatment. Reproduced under the terms of the CC‐BY 4.0 license.^[^ ^144^ ^]^ Copyright 2022, The Authors, Published by AIP Publishing. b) PL spectra of pristine WS_2_, F_4_TCNQ‐doped WS_2_, F_4_TCNQ‐doped WS_2_ after annealing at 450 °C under vacuum for 20 min and F_4_TCNQ‐doped WS_2_ after a second doping round. Reproduced with permission.^[^ ^55^ ^]^ Copyright 2014, American Chemical Society.

Considering the effective modulation of the optoelectronic properties due to the adsorption of common gases, specific organic molecules have also been analyzed as intentional dopants. In this case, molecules are not directly adsorbed in the gas phase. Instead, they are dissolved in a solution in which the WS_2_ samples are immersed and subsequently rinsed.^[^ ^65^, ^144^ ^]^ Hence, the concentration of dopant molecules and the treatment time in the solution are the parameters that control the amount of molecules adsorbed on the MLs.^[^ ^65^ ^]^ For example, penta‐methylrhodocene dimer (RhCpCp^*^)_2_ is a reducing species that can be cleaved in two monomeric cations, releasing two electrons to WS_2_.^[^ ^65^, ^144^ ^]^ The effectiveness of the rise in electron concentration resulting from the adsorption of this donor molecule is confirmed by electric‐transport measurement in FET devices. As visible in the transcharacteristics in **Figure** **17**a, with an increasing time of immersion in the dopant solution, the threshold voltage largely shifts toward negative voltages (e.g., 50 V shift after only 1 min of soaking), similarly to the other n‐dopants. It is possible to obtain an n‐degenerate behavior after 10 min.^[^ ^144^ ^]^ In addition to an effective conductivity increase, the n‐doping is also responsible for a large decrease in the contact resistance at the metal‐WS_2_ interface due to a three‐fold reduction of the Schottky barrier.^[^ ^144^ ^]^ Finally, the modulation of PL due to (RhCpCp^*^)_2_ is in agreement with the increase of the electron concentration upon adsorption (PL decrease due to exciton‐to‐trion conversion).^[^ ^65^ ^]^

Organic molecules with cyano groups, as TCNE (tetra‐cyanoethylene), TCNQ (tetra‐cyanoquino‐di‐methane), and F_4_TCNQ (tetra‐fluoro‐tetra‐cyanoquino‐di‐methane), are acceptor species for WS_2_.^[^ ^55^, ^133^ ^]^ For example, WS_2_ samples treated with F_4_TCNQ are characterized by a decrease in the electron concentration of about one order of magnitude due to the strong electron‐withdrawing ability of the molecule.^[^ ^55^ ^]^ This effect has direct consequences on the performance of F_4_TCNQ‐doped FETs, similarly to other p‐type defects (e.g., Nb_W_, Se_S_, N_S_, and CH_S_), previously discussed. The carrier modulation effects are even more evident in the PL spectra of F_4_TCNQ doped samples.^[^ ^55^ ^]^ As visible in Figure 17b, starting from a pristine spectrum in which there is a unique trion peak, the PL clearly evolves toward an increase in the exciton role in F_4_TCNQ‐doped samples (blue‐shift), due to the decrease of the electron concentration. However, the PL modification due to F_4_TCNQ adsorption is reversible upon annealing at 450 °C.^[^ ^55^ ^]^ The spectra reported in Figure 17b show the possibility of continuously modulating the exciton and trion emission through a series of cycles of doping and annealing. Similar modifications of the optoelectrtonic properties of WS_2_ have been obtained with other acceptor molecules, such as tris‐(4‐bromophenyl)ammoniumyl‐hexa‐chloroantimonate (also known as “Magic Blue”)^[^ ^65^ ^]^ or superacids as TFSI (bis‐(tri‐fluoromethane)sulfonimide).^[^ ^66^ ^]^ In addition to carrier modulation, organic molecules can be used to passivate S vacancies and enhance the material properties.^[^ ^66^, ^145^ ^]^ For example, treatments with oleic acid have been suggested as an effective way to enhance the PL by defect passivation.^[^ ^66^, ^94^ ^]^ A detailed comparison of oleic acid and TFSI superacid treatments reveals several advantages of the first approach.^[^ ^66^ ^]^ The PL enhancement mechanism is different in the two cases. TFSI is an acceptor molecule, so the PL increase is associated with a trion‐to‐exciton conversion. However, this also implies an unavoidable blue‐shift of the peak and a decrease in the electron current in FET devices. On the contrary, oleic acid is not a strong p‐dopant and it increases the PL by V_S_ passivation, minimally altering the position of the PL peak and the n‐type conductivity of the samples. In the latter case, the PL is not limited by trap states and it is possible to get a higher PL increase with respect to the TFSI approach. Moreover, oleic acid, being a weak acid, does not damage the electrode or the experimental apparatus.^[^ ^66^ ^]^ Additionally, the adsorption of oleic acids on WS_2_ forms a hydrophobic barrier on the sample, preventing the uncontrollable effects of O_2_ and H_2_O adsorption.^[^ ^66^ ^]^

Finally, V_S_ passivation and carrier modulation can be achieved with the same molecule (e.g., the organic superacid tri‐fluoromethanesulfonic TFMS^[^ ^145^ ^]^), by properly tuning the functional groups of the dopant species.^[^ ^134^ ^]^

## 6 Conclusions and Future Perspectives

This review provides a systematic overview of the role of different types of defects in WS_2_ MLs, including both intrinsic and intentionally created defects. Experimental evidence and ab initio simulation results are presented to elucidate the impact of these defects on the electronic and optical properties of WS_2_. First, intrinsic defects (e.g., S vacancies, W vacancies, and other foreign impurities such as O and H) are discussed to highlight both negative and positive effects on “as‐synthesized” WS_2_ samples. The analysis of these defects is also correlated with the origin of the n‐type conductivity of WS_2_ MLs. Subsequently, a wide range of intentional defect strategies, including traditional substitution approaches and innovative SCTD by adatom and molecule adsorption, are presented. These approaches demonstrate the possibility to modulate the carrier concentrations and improve the electric‐transport performance of WS_2_‐based devices. The possibility of healing the intrinsic defects with foreign atoms/molecules is also extensively discussed. The modifications of the electronic properties are always correlated with the modulation of the optical properties (i.e., mainly the PL emission spectra). The results of this review are summarized in **Tables** **2** and **3**, in which the discussed defects are classified as intrinsic, isoelectronic substituent (iso. sub.), n/p‐type substituent (n/p‐type sub.), adatom, molecule adsorbed from the gas phase (gas molecule) or organic molecule adsorbed from liquid solution (organic molecule). The main effects on the electronic and optical properties of WS_2_ are also reported.

**Table 2:** Summary of the effects of the different defects on the electronic and optical properties of WS_2_ ML.

Defect	Type	Positive effects	Negative effects	Refs.
V_S_	Intrinsic	Supposed increase of electron density,	Increase of non‐radiative processes,	[12, 32, 40, 54, 56, 57, 60, 72, 73, 75]
		enhancement of the electron mobility,	severe PL suppression	
		red‐shift of the absorption edge,		
		existence of the PL defect‐bound peak for SPE,		
		enhancement of SHG		
O_S_	Intrinsic	Suppression of non‐radiative processes,	Reduction of electron density,	[41, 56, 60, 64]
		blue‐shift and large enhancement of PL peak,	reduction of electron mobility	
		increased stability toward oxidation		
V_W_	Intrinsic		Reduction of electron density,	[57, 60, 62, 73, 81, 83]
			reduction of electron mobility,	
			increase of non‐radiative processes,	
			severe PL suppression	
H impurity	Intrinsic	Increase of electron density		[57, 85, 86]
Mo_W_	Iso. sub.	Effective PL modulation in alloy	Tendency to V_S_ cluster	[38, 101, 102, 103]
Cr_W_	Iso. sub.	Existence of the PL defect‐bound peak for SPE		[38, 48, 59, 68, 100]
Re_W_	n‐type sub.	Existence of the PL defect‐bound peak for SPE	Inefficient n‐dopant	[95]
Cu_W_	n‐type sub.	Improvement of the contact resistance		[18, 108]
Fe_W_	n‐type sub.		Inefficient n‐dopant,	[68, 100, 107]
			PL suppression	
Nb_W_	p‐type sub.	Effective control of carrier polarity,		[57, 91, 92, 100, 111, 112, 146]
		improvement of the contact resistance,		
		red‐shift of PL peak		
V_W_	p‐type sub.	Effective control of carrier polarity	PL suppression	[113]
In_W_	p‐type sub.	Effective control of carrier polarity,		[114]
		PL enhancement		
Se_S_	Iso. sub.	Effective control of carrier polarity in alloy,		[115, 116, 147]
		controllable PL emission in alloy		
Te_S_	Iso.sub.	Controllable properties of alloy	Issues in phase transition for alloy	[117]
Cl_S_	n‐type sub.	Improvement of the contact resistance,		[57, 88, 118, 119, 120, 148]
		passivation of V_S_,		
		red‐shift and enhancement of the PL peak		
N_S_	p‐type sub.	Effective control of carrier polarity,		[50, 51, 52, 67, 93, 122]
		PL enhancement,		
		existence of the PL defect‐bound peak for SPE		
CH_S_	p‐type sub.	Effective control of carrier polarity	PL suppression	[38, 63, 123]

**Table 3:** Summary of the effects of the different adsorbed species on the electronic and optical properties of WS_2_ ML.

Defect	Type	Positive effects	Negative effects	Refs.
Cl	Adatom	Tunable and reversible control of electron density,		[121, 129]
		blue‐shift and enhancement of the PL peak,		
		controllable degree of the light polarization		
F	Adatom	Extremely efficient control of electron density,	Use of fluorine chemistry	[17, 125]
		PL enhancement		
NO_2_	Gas molecule	Efficient p‐dopant,		[139, 140, 141]
		blue‐shift of PL peak		
NH_3_	Gas molecule	N‐dopant in low electron density samples,		[17, 98, 139]
		red‐shift of the PL peak		
H_2_O	Gas molecule	Efficient p‐dopant,		[42, 55, 132, 138]
		blue‐shift and enhancement of the PL peak		
O_2_	Gas molecule	Efficient p‐dopant,	Tendency to oxidation	[27, 53, 62, 132, 138, 142]
		V_S_ passivation,		
		blue‐shift and enhancement of the PL peak		
(RhCpCp^*^)_2_	Organic molecule	Controllable n‐dopant,	PL suppression	[65, 144]
		improvement of the contact resistance		
F_4_TCNQ,“Magic Blue”,TFSI	Organic molecule	Efficient p‐dopant,	Possible corrosion effects	[55, 65, 133, 149]
		blue‐shift and enhancement of the PL peak		
Oleic acid	Organic molecule	V_S_ passivation,		[66, 94]
		PL enhancement,		
		absence of unwanted corrosion,		
		creation of hydrophobic protection barrier		
TFMS	Organic molecule	Efficient p‐dopant,		[145]
		V_S_ passivation		

Despite extensive analyses of defects and adsorbed chemical species in WS_2_, there are still significant challenges to overcome in order to achieve effective defect engineering for technological applications. First, the origin of the intrinsic n‐doping in WS_2_ and its correlation with intrinsic defects must be clarified in an uncontroversial way. Then, considering that intrinsic defects are unavoidable, it is important to characterize the interactions between intentional and intrinsic defects. For instance, a detrimental tendency of S vacancy to cluster in the proximity of some W substituents is observed, as discussed in Section 5.1. Nevertheless, there remains a knowledge gap regarding the effectiveness of doping strategies (both substituent and SCTD) in the presence of intrinsic defects. Therefore, any proposed doping approach must be thoroughly validated by also including intrinsic defects in the analysis. Another possible future field of investigation is related to the combination of different intentional doping strategies. Excluding isolated studies on the co‐presence of different dopant atoms,^[^ ^100^ ^]^ currently most studies analyze doping approaches as mutually exclusive, focusing on the incorporation and characterization of a single type of defect at a time. However, different doping strategies coexist in standard semiconductor technology. For example, dopant ion implantation is used to locally modify the background doping achieved during wafer production and the doping compensation is commonly employed to obtain regions with opposite carrier polarity in the same material. Therefore, a logical next step in the defect engineering of WS_2_ is the study of MLs with a more complex doping profile, where the electronic and optical properties are modulated by the combined use of multiple defect strategies (e.g., the use of SCTD to obtain a spatially selective control of the properties in a WS_2_ ML in which there is a uniform distribution of substituent dopant atoms). This challenge requires the development of mutually compatible defect strategies, the analysis of the possible cross‐talks between different species and a fine control of the concentrations of the different defects to find the optimal balance among the various approaches. This review shows that the diverse range of defect strategies available (W substituent vs S substituent, substituent defect vs SCTD, in‐growth vs post‐growth routes) offers a larger number of degrees of freedom to tune the properties of 2D materials as WS_2_ with respect to 3D semiconductors. Consequently, significant advancements in WS_2_ ML‐based devices can be expected in the near future thanks to a more informed and systematic mastery of the different defect engineering approaches. By leveraging the plethora of available defect strategies, researchers can enhance the performance of WS_2_ MLs and unlock their full potential for a large number of applications.

## Conflict of Interest

The authors declare no conflict of interest.
